# Tuning Luminescent Thermometry in Ytterbium N_3_O_2_ Field-Induced Single-Molecule Magnets via Auxiliary Ligands

**DOI:** 10.1021/acs.inorgchem.6c03015

**Published:** 2026-09-16

**Authors:** Paula Oreiro-Martínez, Julio Corredoira-Vázquez, Ana M. García-Deibe, Javier Cepeda, Enrique Colacio, Federico Totti, Jakob K. Staab, Matilde Fondo

**Affiliations:** † Departamento de Química Inorgánica, 16780 Facultade de Química, Universidade de Santiago de Compostela, Santiago de Compostela 15782, Spain; ‡ Department of Chemistry “U. Schiff”, University of Florence, Sesto Fiorentino 50019, Italy; § Institute of Materials (iMATUS), Universidade de Santiago de Compostela, Santiago de Compostela 15782, Spain; ∥ Departamento de Química Aplicada, 16402 Facultad de Química, Euskal Herriko Unibertsitatea (EHU), Donostia-San Sebastián 20018, Spain; ⊥ Departamento de Química Inorgánica, 16741 Facultad de Ciencias, Universidad de Granada, Avda Fuentenueva s/n, Granada 18071, Spain

## Abstract

The reactions of ytterbium­(III) bromide or iodide with the *N*_3_ *O*_2_ Schiff base 2,6-bis­(2-hydroxyphenyliminomethyl)­pyridine (H_2_L) afford the complexes [Yb­(L)­Br­(CH_3_CH_2_OH)]·CH_3_OH (1·CH_3_OH) and [Yb­(L)­(H_2_O)_3_]­I·CH_3_CH_2_OH·H_2_O (2·CH_3_CH_2_OH·H_2_O), respectively, as single crystals. X-ray diffraction reveals markedly different geometries around the Yb^3+^ ions. Thus, 1 features a seven-coordinate pentagonal-bipyramidal arrangement, whereas 2 exhibits a triangular dodecahedral environment. Magnetic studies indicate that both compounds exhibit field-induced single-ion magnet (SIM) behavior, with magnetization relaxation governed by quantum tunneling of the magnetization (QTM) and Raman processes. Furthermore, temperature-dependent luminescence measurements demonstrate that both complexes can function as nanoscale luminescent thermometers, resulting in bifunctional magnetic–thermometric molecular materials. Notably, the auxiliary ligands exert a pronounced influence on the thermometric performance while only moderately affecting the magnetic behavior. Complex 1 displays superior thermometric properties, reaching a relative sensitivity of 3.7% K^–1^ at 60 K, which represents the highest value reported to date for mononuclear Yb^3+^ field-induced single-ion magnets, together with a minimum temperature uncertainty of 0.2 K. *Ab initio* calculations provide a theoretical interpretation of both the observed magnetic relaxation dynamics and the luminescence properties.

## Graphical abstract

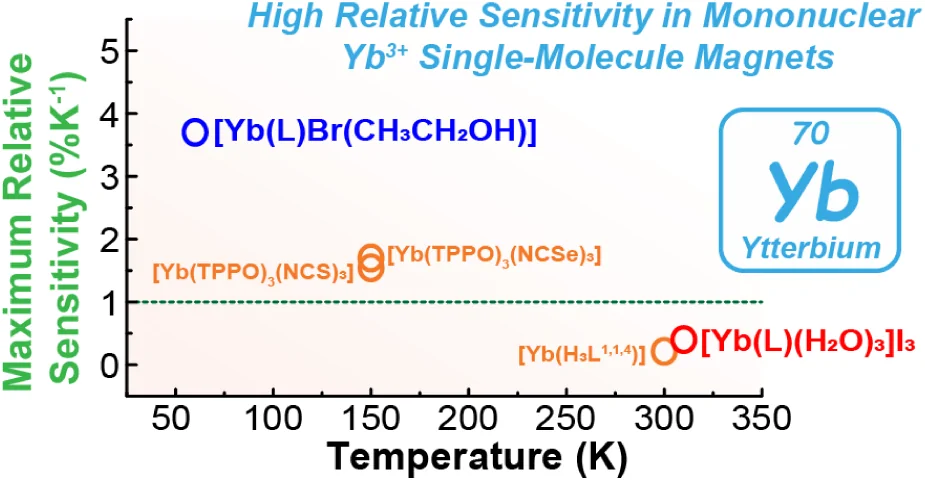

## Introduction

Single-molecule magnets (SMMs) have received growing attention as molecular systems capable of retaining magnetic information at the nanoscale. Their behavior arises from slow relaxation of the magnetization, which in lanthanoid-based complexes is governed by strong spin–orbit coupling and pronounced magnetic anisotropy.^1^ Among these species, Yb^3+^ single ion magnets (SIMs) represent a particularly intriguing subclass. Unlike other lanthanoids commonly employed in high-performance SIMs,^2–5^ Yb^3+^ possesses a 4f^13^ electronic configuration that generates a Kramers ground doublet highly sensitive to subtle variations in the coordination environment.^6^ This sensitivity not only modulates magnetic relaxation pathways, often permitting precise control over Raman and thermally activated Orbach processes, but also gives rise to characteristic near-infrared (NIR) luminescence associated with the ^2^F_5/2_ → ^2^F_7/2_ transition.^7^ The Stark splitting of both the ground and excited multiplets involved in this characteristic NIR transition reflects the local ligand field, rendering Yb-based compounds particularly attractive from a spectroscopic standpoint. As a result, their thermally dependent emission profiles can be accurately analyzed using ratiometric approaches.^8^ These characteristics, together with the sharp NIR emission bands and the minimal interference from visible background signals, make Yb^3+^ one of the most promising lanthanoid ions for luminescence thermometry.

Thus, the coexistence of magnetic anisotropy and well-resolved NIR emission offers a unique opportunity, given that the same Yb^3+^ center responsible for SIM behavior can simultaneously operate as an intrinsic luminescent thermometer. Despite this appealing duality, systems combining these two functions remain scarce. To date, only a limited number of field-induced single-ion Yb^3+^ magnets have been reported to exhibit luminescent thermometry, including three Yb^3+^ mononuclear systems,^9,10^ in addition to a few heterometallic Yb^3+^/Co^3+^ and Yb^3+^/Cu^+^ complexes,^11–13^ a homodinuclear Yb_2_ single-molecule magnet,^8^ and three heterodinuclear Nd^3+^/Yb^3+^ systems.^13^ This limited family of compounds underscores the difficulty of achieving Yb^3+^ materials in which magnetic bistability and efficient thermometric emission coexist within a single molecular scaffold.

Accordingly, the development of these bifunctional Yb^3+^-compounds requires the rational design of ligands capable of simultaneously improving both properties. Building on our previous experience with Schiff base ligands, we selected a previously reported rigid, dianionic pentadentate *N*_3_ *O*_2_ ligand. Its strong in-plane charge donation is expected to stabilize the crystal field required to enhance the magnetic anisotropy of the prolate Yb^3+^ ion, while its conjugated pyridine–imine framework should efficiently sensitize the characteristic near-infrared emission through the antenna effect.

On this basis, we report here two Yb^3+^ complexes that successfully combine single-ion magnet behavior with luminescence thermometry. Furthermore, the influence of the auxiliary ligands on both the field-induced magnetic relaxation and the thermometric performance is investigated.

## Experimental Section

### General

All reagents employed in the synthesis were obtained from commercial suppliers (Merck and Sigma-Aldrich), had a purity higher than 99%, and were used as received without further purification. Absolute ethanol (98% purity) was purchased from Merck. C, H, and N contents were determined using a Thermo Scientific FlashSmart elemental analyzer. Infrared characterization was carried out with a Varian FT-IR 670 spectrometer fitted with an attenuated total reflectance (ATR) accessory, collecting spectra in the 400–4000 cm^–1^ region.

### Syntheses

The H_2_L ligand used in this work was previously reported.^9,14,15^

**[Yb­(L)­(Br)­(CH**_**3**_ **CH**_**2**_ **OH)]** (**1**): An absolute ethanol (10 mL) solution of YbBr_3_·9H_2_O (0.181 g, 0.315 mmol) was added to an absolute ethanol (35 mL) solution of H_2_L (0.100 g, 0.315 mmol) and Et_3_N (0.064 g, 0.630 mmol). The reaction mixture was maintained under continuous stirring at ambient temperature for 3 h, and subsequently filtered. The slow evaporation of a mixture of the mother liquor with added methanol yielded single crystals of [Yb­(L)­(Br)­(CH_3_CH_2_OH)]·CH_3_OH (**1**·CH_3_OH), of suitable quality for single-crystal X-ray diffraction analysis. The crystalline material was isolated by filtration and dried in a laboratory oven, where the MeOH solvent was removed to afford compound **1**. Yield: 111 mg (57%). Elemental analysis calcd. for C_21_H_19_BrN_3_O_3_Yb (614.33): C 41.05, N 6.84, H 3.12%. Found: C 40.93, N 6.75, H 3.23%. IR (ATR, ν̃ /cm^–1^): 1533 (*v*CN_py_), 1583 (*v*CN_imine_), 3060 (*v*OH).

**[Yb­(L)­(H**_**2**_ **O)**_**3**_ **]­I·CH**_**3**_ **CH**_**2**_ **OH·H**_**2**_ **O** (**2·**CH_3_CH_2_OH·H_2_O): An absolute ethanol (10 mL) solution of YbI_3_·9H_2_O (0.226 g, 0.315 mmol) was added to an absolute ethanol (35 mL) solution of H_2_L (0.100 g, 0.315 mmol) and Et_3_N (0.064 g, 0.630 mmol). The reaction mixture was maintained under continuous stirring at ambient temperature for 3 h, and subsequently filtered. Single crystals of [Yb­(L)­(H_2_O)_3_]­I·CH_3_CH_2_OH·H_2_O, of sufficient quality for single-crystal X-ray diffraction analysis, were obtained upon gradual evaporation of the remaining solution. The crystals were collected by filtration and then dried in a laboratory oven. Yield: 149 mg (64%). Elemental analysis calcd. for C_21_H_27_IN_3_O_7_Yb (733.39): C 34.39, N 5.72 H 3.71%. Found: C 34.49, N 5.67, H 3.83%. IR (ATR, ν̃ /cm^–1^): 1533 (*v*CN_py_), 1583 (*v*CN_imine_), 3220 (*v*OH).

### Single X-ray Crystallographic Refinement and Structure Solution

Single crystals of **1**·CH_3_OH and **2**·CH_3_CH_2_OH·H_2_O could be obtained as detailed above. Table S1 compiles the principal crystallographic parameters together with the refinement details. Data were collected at 293 K for **1**·CH_3_OH and 100 K for **2**·CH_3_CH_2_OH·H_2_O on a Bruker D8 VENTURE PHOTON III-14 diffractometer, employing graphite monochromatized Mo-*K*α (λ = 0.71073 Å) radiation. Absorption effects were corrected using a multiscan procedure implemented in the SADABS program.^16^ The crystal structures were solved using conventional direct methods employing SHELXT,^17^ and subsequently refined with SHELXL,^18^ from the program package SHELX-2018, by full matrix least-squares procedures based on *F* ^2^. All non-hydrogen atoms were refined with anisotropic displacement parameters. Carbon-bound hydrogen atoms were introduced at calculated geometrical positions and included in the structure-factor calculations. In contrast, hydrogen atoms bonded to N or O were identified from the corresponding Fourier difference maps in order to establish the hydrogen-bonding interactions. These atoms were either refined without constraints or assigned displacement parameters derived from those of the corresponding parent atoms. CCDC 2543031–2543032 contain the supplementary crystallographic data for this paper. These data can be obtained free of charge from the Cambridge Crystallographic Data Centre via www.ccdc.cam.ac.uk/data_request/cif.

### Powder X-ray Diffraction Studies

Powder X-ray diffraction data for **2**·CH_3_CH_2_OH·H_2_O were acquired using a Philips diffractometer comprising a PW1710 control unit, a PW1820/00 vertical goniometer, and an Enraf Nonius FR590 generator operated at 40 kV and 30 mA. CuKα (1.5406 Å) radiation was generated with a sealed copper tube and selected using a graphite monochromator. Measurements were performed over the 2θ interval from 2 to 30°, using 0.02° increments and a 3 s acquisition time at each step. During data collection, the sample was continuously rotated to improve the quality of the diffraction profiles and reduce preferred-orientation effects.

For sample mounting, an oriented Si(511) crystal plate was employed as the substrate, thereby minimizing background scattering associated with conventional glass holders. Prior to measurement, the material was finely ground in a mortar to further reduce any contribution from preferential crystallite orientation. The resulting diffraction patterns were subsequently processed and analyzed with HighScore Plus software, version 3.0d.

### Magnetic Measurements

The magnetic response of **1** and **2**·CH_3_CH_2_OH·H_2_O was investigated through both *dc* and *ac* susceptibility measurements using a PPMS Quantum Design susceptometer. *dc* magnetic susceptibility measurements were recorded in the temperature range 2–300 K under a magnetic field of 1000 Oe. Magnetization measurements at 2.0 K were recorded under magnetic fields ranging from 0 to 70000 Oe. Diamagnetic contributions were corrected using Pascal’s Tables. *ac* susceptibility experiments were performed in the 2–15 K temperature interval by applying a 1500 Oe static field together with a 3 Oe oscillating field, using frequencies between 70 and 10000 Hz.

### Spectroscopic Characterization

Solid-state absorption measurements were performed with a Shimadzu UV-2600 UV-Vis spectrophotometer. Photoluminescence (PL) lifetimes and steady-state emission spectra were recorded for crystalline samples between 10 K and room temperature using an Edinburgh Instruments FLS920 spectrometer equipped with a closed-cycle helium cryostat. Excitation was provided by an IK3552R-G He–Cd continuous-wave laser operating at 325 nm. The emitted radiation in the visible region was detected with a photomultiplier tube (PMT) coupled to the spectrometer, whereas NIR emission was monitored using a Hamamatsu NIR-PMT H10330C-75 detector operated at a voltage of 850 V. On the other hand, a PLS-340 pulsed led of PicoQuant (λ = 340 nm) was used as excitation source for recording decay curves in the visible spectrum, using the previously mentioned setup, whereas an Ekspla NT-342B-SH-AW pulsed laser was employed for decay curves in the NIR range (Yb^3+^-centered ^2^F_5/2_ → ^2^F_7/2_ emission).

### Luminescence Thermometry

A spectral deconvolution procedure using OriginLab© software was implemented to calculate the integrated areas of the Stark splitting of the Yb^3+^ (^2^F_5/2_ → ^2^F_7/2_) transition for **1** and **2**·CH_3_CH_2_OH·H_2_O. First, baseline correction was performed using the baseline subtraction tools available in Origin. Subsequently, the spectra were transformed from wavelength to energy scale to ensure a consistent evaluation of the emission intensities across the entire spectral range using

S1 $$E=\frac{10^{7}}{\lambda }$$

where *E* denotes the energy, expressed in cm^–1^, and *λ* represents the associated wavelength in nm. However, the signal value recorded from the spectrometer is a signal per unit wavelength. Because wavelength and energy are inversely related, the *dλ* interval in the wavelength spectrum has different *dE* sizes across the entire energy spectrum. Therefore, based on energy conservation, the measured intensity must be corrected according to the following expression:

S2 $$f\left(E\right)=f\left(\lambda \right)\frac{d\lambda }{dE}=f\left(\lambda \right)\frac{d}{dE}\left(\frac{10^{7}}{E}\right)=-f\left(\lambda \right)\frac{10^{7}}{E^{2}}$$

where *f*(*E*) and *f*(*λ*) denote the emission intensities expressed as functions of energy and wavelength, respectively. This relationship corresponds to a Jacobian transformation and the negative sign can be disregarded because it simply reflects the opposite directions of integration in wavelength and energy.^19,20^ The wavelength-to-energy conversion is especially relevant for spectra containing several emission features or broad bands extending over a wide spectral region.

To quantitatively analyze the emitter populations and understand the cause of the line width, the Jacobian-corrected spectra should be further processed to extract information about the emitter populations from their relative peak areas. The process involves converting the spectra into specific line shapes by factoring out the *E* ^3^ dependence of spontaneous emission as^21–23^

S3 $$\frac{f\left(E\right)}{E^{3}}=-f\left(\lambda \right)\frac{10^{7}}{E^{5}}$$

Finally, the spectra were deconvoluted using multicomponent fitting procedures. In the case of compound **1**, the spectrum was deconvoluted using six components, whereas four components were employed for **2**·CH_3_CH_2_OH·H_2_O, as described in the main article.

For the calculation of the thermometric parameters, Δ corresponds to Δ_1_ for compound **1** and Δ_2_ for compound **2**·CH_3_CH_2_OH·H_2_O, as defined in the main text.

In the case of compound **1**, the thermometric parameter Δ_1_ was calculated from the integrated areas of the deconvoluted Gaussian peaks. Specifically, *I*_1_ corresponds to the sum of the integrated areas of the four Gaussian components centered at 1048, 1029, 1020, and 970 nm, whereas *I*_2_ corresponds to the sum of the integrated areas of the two Gaussian components centered at 992 and 1003 nm. For compound **2**·CH_3_CH_2_OH·H_2_O, a custom MATLAB routine was developed to determine the integrated areas of the emission spectra. The thermometric parameter was then calculated from the integrated emission intensities, where *I*_1_ and *I*_2_ correspond to the integrated intensities in the 950–980 nm and 980–1000 nm spectral regions, respectively.

The performance of luminescent thermometers is commonly assessed using two key figures of merit: the relative thermal sensitivity (*S_r_*) and the temperature uncertainty, or temperature resolution (*δT*).^24,25^ These parameters provide useful metrics for comparing thermometric performance across systems with different chemical compositions and environments.^26^ The thermal relative sensitivity (*S_r_*) is defined as

S1 $$S_{r}=\frac{1}{\Delta }\left|\frac{\partial \Delta }{\partial T}\right|$$

On the other hand, *δT* is expressed as

S2 $$\delta T=\frac{1}{S_{r}}\frac{\delta \Delta }{\Delta }$$

where *δ*Δ/Δ represents the relative uncertainty associated with the determination of the thermometric parameter. The uncertainties in the integrated areas of these bands (*δI_i_*) were determined from the emission spectra by calculating the signal-to-noise ratio (SNR), defined as the ratio between the measured signal intensity and the corresponding noise level.

S3 $$\delta I_{i}=\frac{1}{SNR}I_{i}$$

The uncertainty in Δ is defined as

$$\delta \Delta =\Delta \times \sqrt{{\left(\frac{\delta I_{1}}{I_{1}}\right)}^{2}+{\left(\frac{\delta I_{2}}{I_{2}}\right)}^{2}}$$

### Computational Details

The molecular structures of **1** and the cation of **2** were extracted from their respective crystal structures. All calculations were performed using ORCA 6.0.^27^ Scalar relativistic effects were included via the Douglas–Kroll–Hess (DKH) method.^28^ The electronic structure was first described at the Complete Active Space Self-Consistent Field (CASSCF)^29^ level using an active space of 13 electrons in 7 orbitals, CAS­(13,7), including seven spin doublets in the state-average. On top of the multireference CASSCF reference, dynamic correlation effects were treated using second-order N-electron valence state perturbation theory (NEVPT2).^30^ In addition, the quasi-degenerate formulation of strongly contracted NEVPT2 (QD-NEVPT2)^31^ was employed to account for interactions between near-degenerate electronic states mixing the CASSCF states. Spin–orbit coupling (SOC) described by an atomic mean-field operator was included using quasi degenerate perturbation theory,^32^ as implemented in ORCA, by mixing all 14 spin states. The resulting spin–orbit coupled states were used to compute magnetic properties, including g-tensors and the matrix elements of the magnetic dipole operator. All calculations employed ANO-RCC^33^ basis sets: VTZP for Yb, VDZP for N, O and Br, and VDZ for C and H. Auxiliary basis sets were generated automatically (AutoAux)^34^ and used within the resolution-of-identity approximation for Coulomb and exchange integrals (RI-JK).^35^

## Results and Discussion

### Synthesis and Structural Characterization

[Yb­(L)­Br­(CH_3_CH_2_OH)]·CH_3_OH (**1**·CH_3_OH) and [Yb­(L)­(H_2_O)_3_]­I·CH_3_CH_2_OH·H_2_O (**2**·CH_3_CH_2_OH·H_2_O) were obtained as single crystals by reaction of the corresponding ytterbium­(III) halide and reported H_2_L (2,6-bis­(2-hydroxyphenyliminomethyl)­pyridine),^14,15,36^ as shown in Scheme 1. The crystals of **1**·CH_3_OH lose the methanol solvate on drying to yield **1**.

**Scheme 1:**
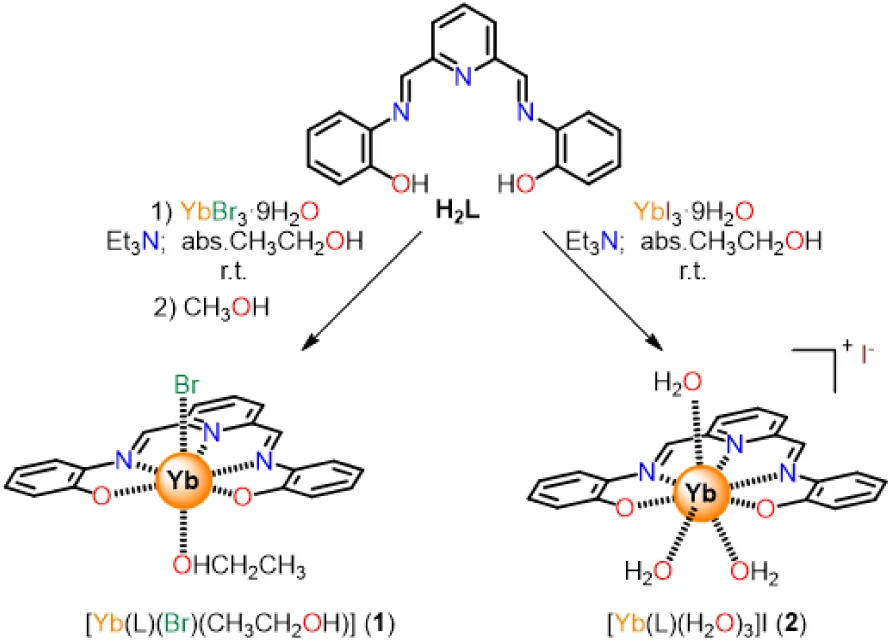
Reaction Scheme for Isolation of [Yb­(L)­Br­(CH_3_CH_2_OH)]·CH_3_OH (**1**·CH_3_OH) and [Yb­(L)­(H_2_O)_3_]­I·CH_3_CH_2_OH·H_2_O (**2**·CH_3_CH_2_OH·H_2_O)^a^

a Solvates are omitted for clarity.

A comprehensive characterization of the complexes was carried out using elemental analysis, infrared spectroscopy, and X-ray diffraction techniques. A detailed magnetic study of the samples was also conducted, with a particular focus on their single-molecule magnet behavior.

The infrared spectra of the compounds (Figure S1) display two prominent bands at 1583 and 1533 cm^–1^, which can be attributed to the *v*(C = N) stretching modes of the imine and pyridine groups,^15,36^ respectively. Both bands are shifted to lower wavenumbers compared with those of the uncoordinated ligand, supporting the involvement of both imine and pyridine nitrogen atoms in coordination to the metal center. Moreover, for **2**·CH_3_CH_2_OH·H_2_O a broad absorption centered around 3220 cm^–1^ is observed, consistent with the presence of O–H groups arising from coordinated and/or solvated water as well as ethanol solvate. In contrast, compound **1** exhibits a narrower band at 3060 cm^–1^ which can be attributed to the O–H stretching vibration of the coordinated ethanol ligand.

### X-ray Diffraction Studies

Experimental details of data acquisition and resolution of single crystals of **1**·MeOH and **2**·CH_3_CH_2_OH·H_2_O are summarized in Table S1. Ellipsoid diagrams for **1** and **2** are shown in Figures 1 and 2, and selected bond lengths and angles are compiled in Tables S2 and S3.

**Figure 1:**
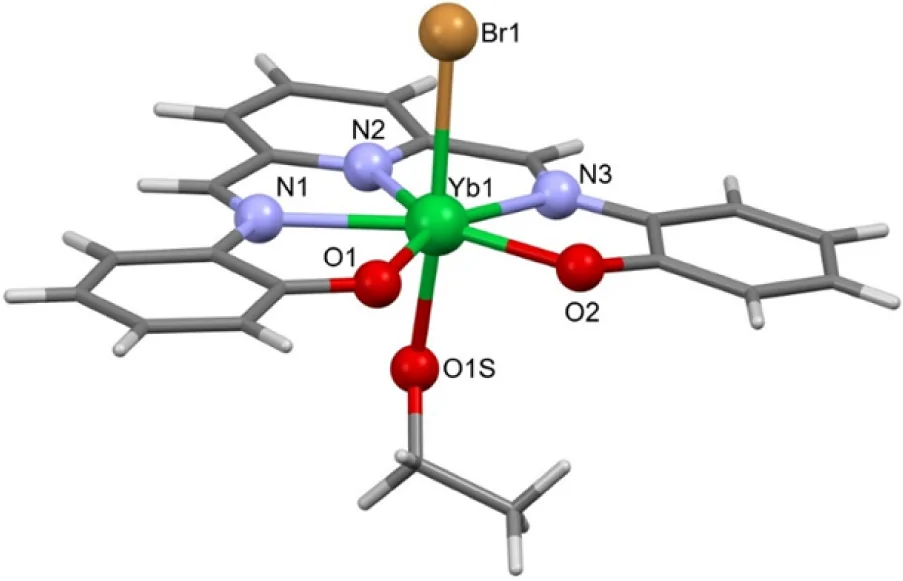
Balls and sticks diagram for [Yb­(L)­Br­(CH_3_CH_2_OH)] in **1**·CH_3_OH. Only the metal ion and heteroatoms of the coordination sphere are labeled.

**Figure 2:**
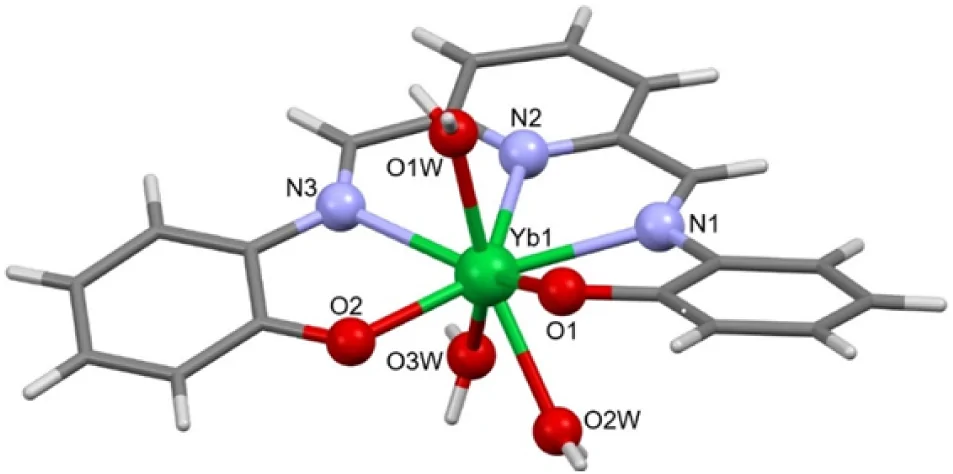
Balls and sticks diagram for the cation [Yb­(L)­(H_2_O)_3_]^+^ in **2·**CH_3_CH_2_OH·H_2_O. Only the metal ion and heteroatoms of the coordination sphere are labeled.

The asymmetric unit of **1**·CH_3_OH contains a neutral [Yb­(L)­Br­(CH_3_CH_2_OH)] molecule and one methanol as solvate. In the [Yb­(L)­Br­(CH_3_CH_2_OH)] complex, both phenolate oxygen atoms of the Schiff base are deprotonated. The resulting dianionic L^2–^ ligand acts as a pentadentate *N*_3_ *O*_2_ donor, using all its donor atoms to link the metal center. This polydentate donor fills the equatorial plane, and it adopts an almost entirely planar arrangement, the largest displacement of an individual atom relative to the calculated mean *N*_3_ *O*_2_ plane being 0.010 Å, with the Yb^3+^ ion deviating 0.136 Å from the plane.

The coordination sphere around the metal center is completed by two additional monodentate ligands, namely an ethanol molecule and a bromide ion. As a result, the Yb^3+^ ion has coordination number 7, with an *N*_3_ *O*_3_ *Br* donor set. Analysis of the distortion of the *YbN*_3_ *O*_3_ *Br* core with respect to an ideal polyhedron of seven vertexes using the SHAPE program^37^ reveals a quite perfect pentagonal bipyramid geometry (Table S4), with an O1S–Yb1–Br1 angle of 165.29(4)°. The remaining bond lengths and angles are consistent with the values typically expected for this type of coordination environment and therefore require no further discussion.^9^ However, it should be noted that the shortest distances around the metal ion are the Yb–O_phenolate_ ones (Yb–O1 and Yb–O2), and the longest one corresponds to the Yb1–Br1 bond.

In this structure, it is also remarkable that the crystallization methanol molecule forms hydrogen-bond interactions involving the coordinated ethanol and the phenolic oxygen atom O1 of two neighboring molecules (Table S2), giving rise to a zigzag chain (Figure S2), with Yb···Yb distances of 8.431(1) Å.

In contrast to complex **1**, which is neutral, **2·**CH_3_CH_2_OH·H_2_O is ionic, a distinction that should be expected to impact its magnetic properties. Thus, its asymmetric unit contains a mononuclear [Yb­(L)­(H_2_O)_3_]^+^ cation (Figure 2), an iodide anion, as well as a water and an ethanol molecule as solvate.

In the [Yb­(L)­(H_2_O)_3_]^+^ cation, the bisdeprotonated Schiff base acts as a pentadentate *N*_3_ *O*_2_ donor, as in **1**. However, the plane conformed by this ligand is much more distorted than in **1**, with distances of the donor atoms to the mean calculated *N*_3_ *O*_2_ plane greater than 0.2 Å for the imine nitrogen atoms, and 0.33 Å for the oxygen atoms (0.335 and 0.361 for O2 and O1, respectively), with one of them above and the other below the mean plane. The ytterbium atom protrudes 0.330 Å from the plane, and its coordination sphere is completed by 3 water molecules, two located on one side of the plane and the other one on the other side. Consequently, the Yb^3+^ center adopts an eight-coordinate environment defined by three nitrogen and five oxygen donor atoms (*N*_3_ *O*_5_). According to the SHAPE analysis,^37^ the *YbN*_3_ *O*_5_ coordination sphere reveals a triangular dodecahedron geometry distorted to biaugmented trigonal prism geometry (Table S4). Again, the bond distances and angles are within norm,^9^ and the shortest distances correspond to the Yb–O_phenolate_ bonds, the longest ones being associated with the Yb–N bonds.

Finally, the H-bond scheme in **2·**CH_3_CH_2_OH·H_2_O (Table S3) is more intricate than in **1**·CH_3_OH. Thus, the three coordinated water molecules, the phenolate oxygen atoms and the crystallization water molecule are implicated to also construct a zigzag chain (Figure S3), but in this case the Yb···Yb distances are notably shorter, of 5.6405(5) Å.

Powder X-ray diffraction measurements for **2·**CH_3_CH_2_OH·H_2_O (Figure S4) are consistent with a highly pure crystalline material, as the experimental diffractogram contains no detectable reflections attributable to secondary phases or impurities. The powder diffractogram for **1** was not recorded as the crystals contain a methanol solvate (**1**·CH_3_OH) that is lost on drying, thus leading to changes on the packing that results in changes in the diffractogram.

### Magnetic Properties

Magnetic susceptibilitymeasures were collected for **1** and **2·**CH_3_CH_2_OH·H_2_O in the range 2–300 K. Figure 3 presents the χ_M_ *T vs T* plots for the two complexes. The χ_M_ *T* values at 300 K are 2.41 cm^3^ K mol^–1^ for **1** and 2.75 cm^3^ K mol^–1^ for **2·**CH_3_CH_2_OH·H_2_O, which are consistent with the magnetic response anticipated for a single Yb^3+^ ion at room temperature (2.57 cm^3^ K mol^–1^). A progressive reduction in χ_M_ *T* is observed upon lowering the temperature, with values of 1.95 cm^3^ K mol^–1^ for **1** and 1.52 cm^3^ K mol^–1^ for **2·**CH_3_CH_2_OH·H_2_O reached at 2 K. The observed decrease is mainly associated with the progressive thermal depopulation of the excited Kramers doublets, together with the significant magnetic anisotropy of the Yb^3+^ center.^9^

**Figure 3:**
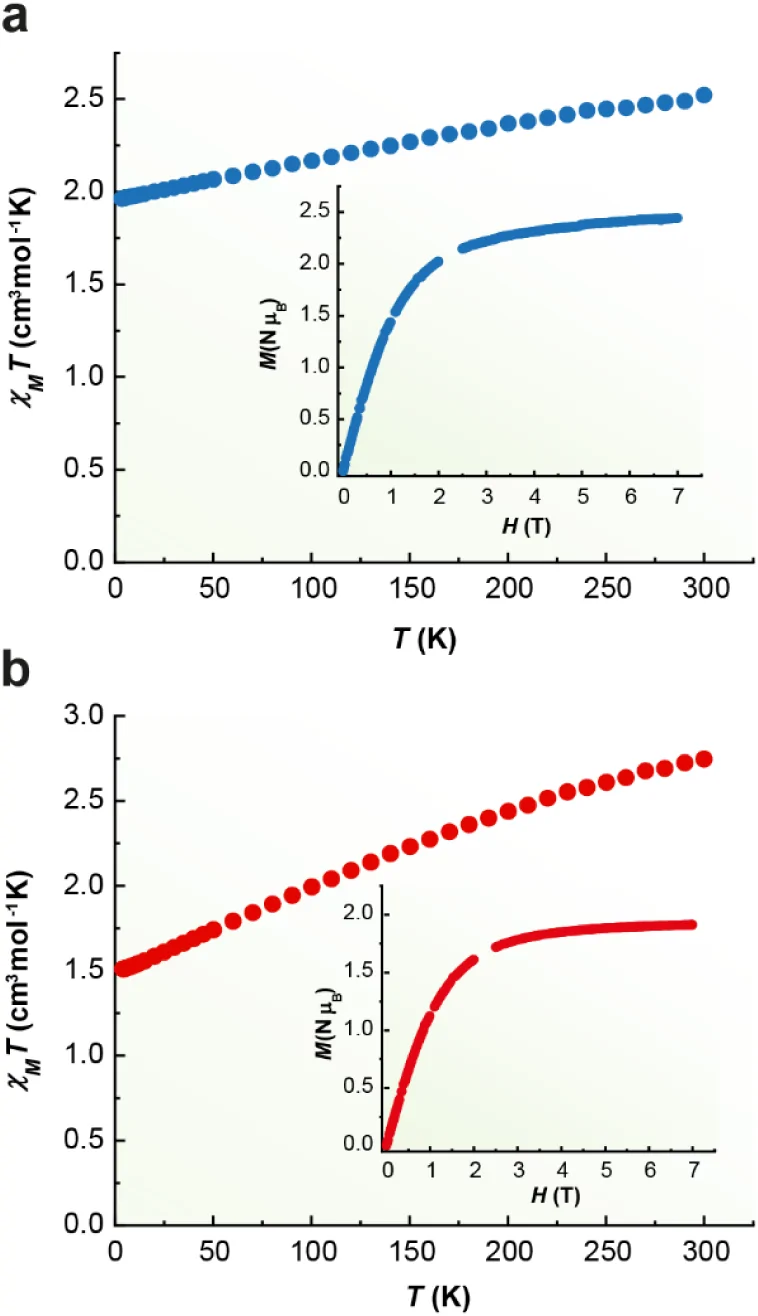
χ_M_ *T vs T* for: **1** (a) and **2·**CH_3_CH_2_OH·H_2_O (b) Insets: *M*/Nμ_B_ *vs H* at 2 K.

Further evidence for this anisotropic behavior is provided by the field-dependent magnetization measurements performed at 2 K over a field range of 0–7 T. At 7 T, the reduced magnetization reaches values of 2.44 Nμ_B_ for **1** and 1.91 Nμ_B_ for **2**·CH_3_CH_2_OH·H_2_O (Figure 3 inset). These values are well below the 4 Nμ_B_ expected for an isolated Yb^3+^ ion, supporting the presence of substantial magnetic anisotropy in both compounds.

To investigate the magnetization relaxation dynamics of compounds **1** and **2·**CH_3_CH_2_OH·H_2_O, *ac* susceptibility measurements were initially carried out under zero applied *dc* field. In neither case was a maximum detected in the out-of-phase component (*χ″*_*M*_) above 2 K, suggesting that fast relaxation through quantum tunneling of magnetization (QTM) may hinder the observation of single-molecule magnet behavior. To minimize the contribution of QTM, additional measurements were therefore performed under a weak static magnetic field.

Thus, new *ac* data for both compounds were recorded under an optimum field of 1500 Oe (Figure S5). In these conditions, **1** and **2·CH_3_CH_2_OH·H_2_O**exhibit a clear dependence of their out-of- phase *ac* susceptibility components on both temperature and frequency. In particular, *χ″*_*M*_ displays frequency-dependent maxima within the 2–7 K range (Figure 4), providing evidence for slow magnetic relaxation and field-induced single-molecule magnet (SMM) behavior in both compounds.

**Figure 4:**
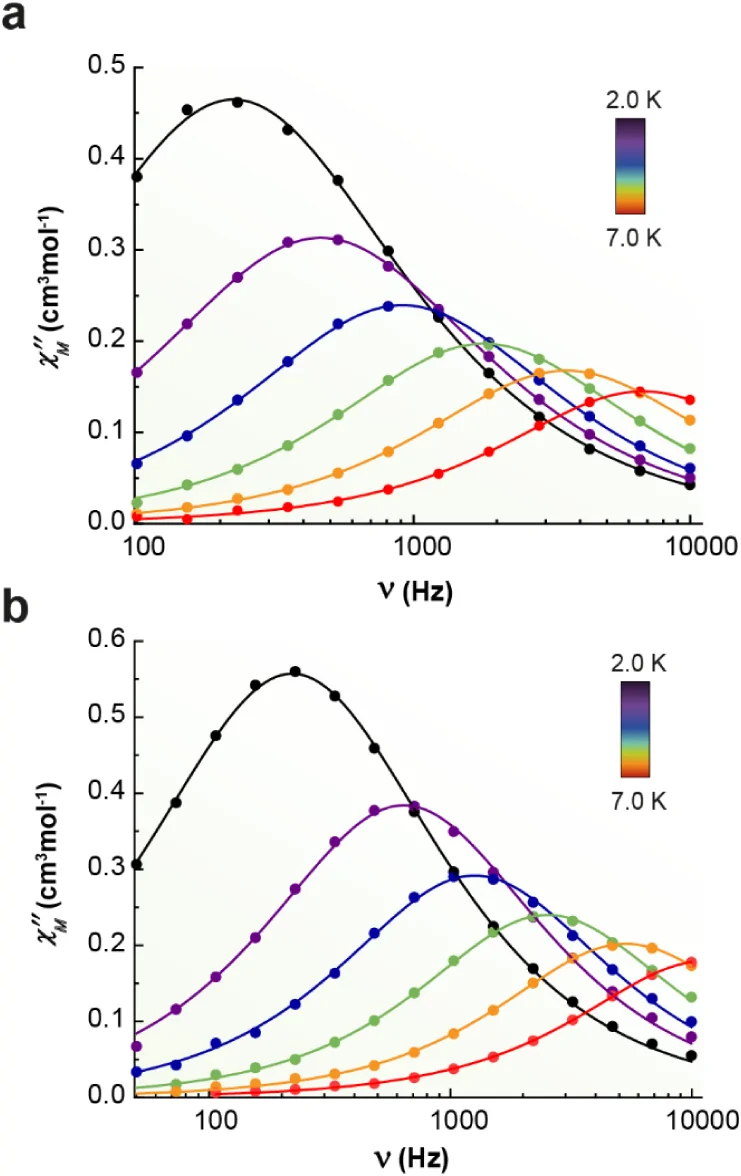
Frequency dependence of *χ″*_M_ for **1** (a) and **2·**CH_3_CH_2_OH·H_2_O (b) in an external *dc* field of 1500 Oe at different temperatures.

Moreover, the magnetization relaxation dynamics were further investigated by analyzing the temperature dependence of the relaxation time (τ) (Figure 5). The α parameters obtained from the Cole–Cole graph (Figure S6) indicate a wider distribution of the relaxation times for **2**·CH_3_CH_2_OH·H_2_O. Besides, the *χ″*_M_ *vs T* graphs (Figure S7) reveal that *χ″*_M_ does not decay to zero at low temperatures for any complex, evidencing incomplete suppression of QTM despite the application of the optimal external field.

**Figure 5:**
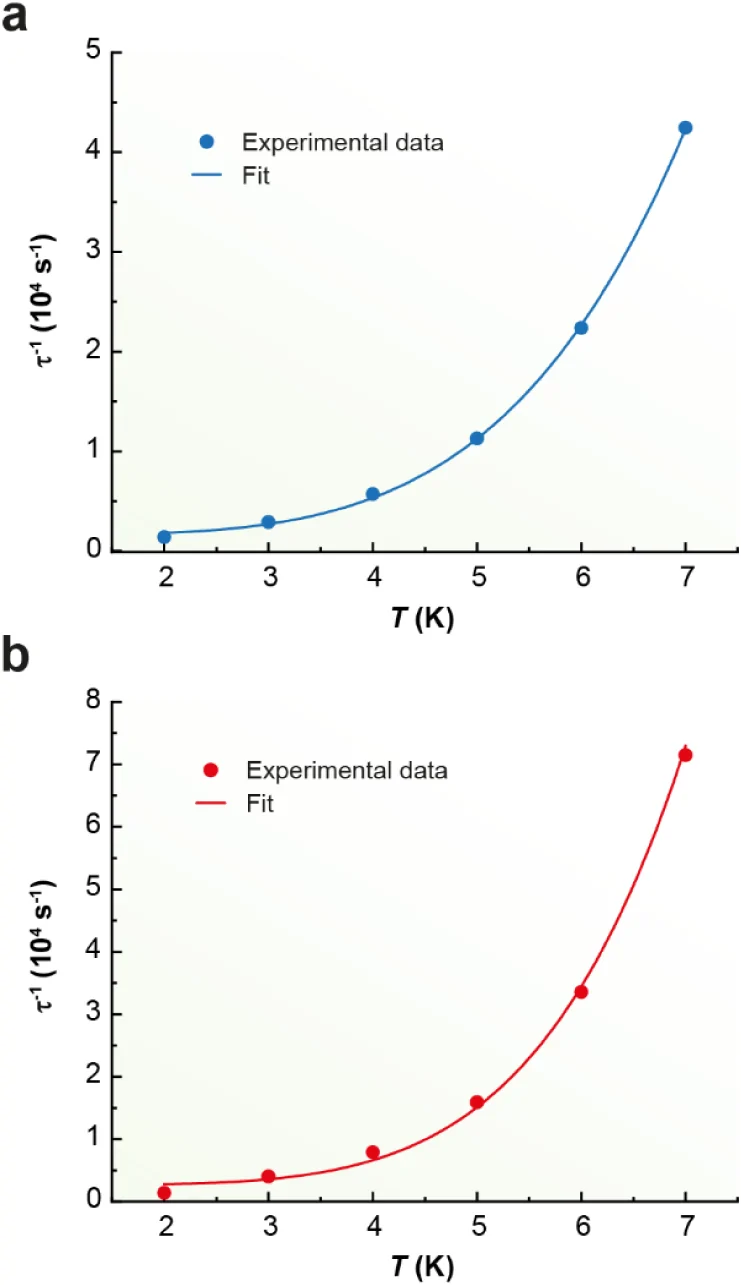
Dependence of the relaxation time with temperature for **1** (a) and **2**·CH_3_CH_2_OH·H_2_O (b) in an external dc field of 1500 Oe. The blue and red solid lines account for the best fit considering Raman plus QTM relaxation processes.

Consequently, extensive efforts were undertaken to model the experimental data by accounting for the different relaxation mechanisms that may contribute to the overall magnetic relaxation (Orbach, Direct, Raman and QTM). Thus, the fit that best replicated these plots considering all these processes (eq 7), individually or grouped, is the one that takes into account Raman plus QTM relaxations.

1 $$\tau^{-1}={\tau_{0}}^{-1}e^{-U_{\mathrm{eff}}/k_{B}T}+\mathrm{AT}+{\mathrm{CT}}^{n}+{\tau_{\mathrm{QTM}}}^{-1}$$

where the first term corresponds to Orbach, the second to direct, the third to Raman and the fourth to QTM relaxation.

This fit for 1 yields the parameters C = 9.8(2) K^–n^ s^–1^, n = 4.3 and τ_QTM_ = 6.1(1) × 10^–4^ s (*R* ^2^ = 0.9996), while for **2·**CH_3_CH_2_OH·H_2_O C = 3.1(1) K^–n^ s^–1^, n = 5.1(1) and τ_QTM_ = 3.7(1) × 10^–4^ s (*R* ^2^ = 0.9989).

For Kramers ions, the theoretical value of *n* is 9; therefore, the value obtained from the fitting may appear relatively low. However, the involvement of both optical and acoustic phonons can lead to lower exponents, typically within the range of 1–6, depending on the specific energy-level scheme.^38^ Accordingly, n values close to 4 have been published for other ytterbium field-induced single ion magnets.^9,39^ When both Orbach and QTM processes are considered, slightly inferior fittings are obtained, yielding the following parameters: *U*_*eff*_ = 21.4(1) K, τ_0_ = 1.21(1) × 10^–6^ s and τ_QTM_ = 8.5(3) × 10^–4^ s (*R* ^2^ = 0.9984) for **1**, and *U*_*eff*_ = 31.8(3) K, τ_0_ = 1.6(1) × 10^–7^ s and τ_QTM_ = 2.7(8) × 10^–4^ s (*R* ^2^ = 0.9949) for **2·**CH_3_CH_2_OH·H_2_O.

This could indicate a slightly higher energy barrier for **2·**CH_3_CH_2_OH·H_2_O than for **1**. However, the high difference in the energy barriers determined from *ac* measurements and *ab initio* calculations (about 490 cm^–1^, *vide infra*) rules out the thermally activated Orbach relaxation pathway.

Accordingly, the magnetic behavior of both complexes is broadly similar, with relaxation governed by QTM and Raman processes and absence of open hysteresis. However, phonon-assisted relaxation (Raman process) is more efficient in **1**, whereas QTM is more pronounced in **2·**CH_3_CH_2_OH·H_2_O. Despite these differences in the dominant relaxation pathways, the overall magnetic response seems to be only marginally affected by the variations in coordination geometry, auxiliary ligands, and charge of the complex.

### Spectroscopic Characterization

The spectroscopic properties of the complexes **1** and **2·**CH_3_CH_2_OH·H_2_O were investigated by means of excitation and emission measurements in the UV-vis-NIR range. A comparative analysis of the emission spectra of both compounds measured under the same conditions (Figure S8), excited under UV light (λ = 325 nm), revealed a similar pattern with significantly more intense NIR emission for complex **1**. The solid-state UV-vis absorption spectra (Figure S9) of both compounds are nearly identical, displaying intense absorption bands below 600 nm assigned to ligand-centered and/or charge-transfer transitions. These results confirm that the L^2–^ ligand acts as an efficient antenna for Yb^3+^ sensitization in both complexes, indicating that the different emission intensities are unlikely to arise from differences in light absorption or energy transfer efficiency. The lower NIR emission intensity observed for **2**·CH_3_CH_2_OH·H_2_O compared to **1** can therefore be reasonably attributed, at least in part, to the presence of coordinated water in **2·**CH_3_CH_2_OH·H_2_O, as it is well-known that this causes efficient deactivation of the Ln^3+^ excited state, via non-radiative energy transfer to vibrational modes of the O–H groups.^40^ This hypothesis is reinforced by the slightly shorter lifetime recorded for the main NIR emission line (λ_em_ = 970 nm) in compound **2·**CH_3_CH_2_OH·H_2_O than in **1** (of 1.03(1) *vs* 1.28(1) μs at room temperatureFigure S10). Nevertheless, the influence of other structural and electronic factors, including differences in coordination geometry and crystal field splitting, cannot be ruled out. Consequently, the spectroscopic discussion presented below is mainly focused on compound **1**.

The low-temperature emission spectrum recorded under excitation at 325 nm exhibits an intense band in the 960–1100 nm region, corresponding to the characteristic ^2^F_5/2_ → ^2^F_7/2_ transition of the Yb^3+^ ion. In addition, a weak contribution arising from residual ligand emission can be observed in the visible region, consisting of a main band centered at *ca*. 370 nm, accompanied by a shoulder at 415 nm and a weak broad emission centered around 630 nm. In contrast, no detectable ligand-centered emission was observed in the visible region for complex **2·**CH_3_CH_2_OH·H_2_O­(Figure 6).

**Figure 6:**
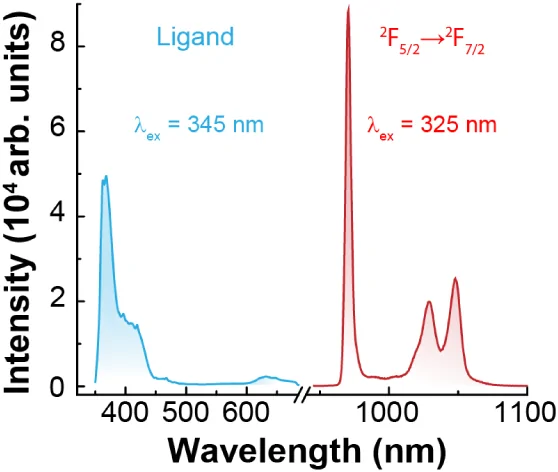
Solid-state spectroscopic measurements of complex **1** recorded at 15 K. The ligand-centered emission spectrum (blue) was recorded under excitation at 345 nm, while the NIR emission spectrum (red) was obtained under excitation at 325 nm. The intense emission band around 980 nm is assigned to the characteristic ^2^F_5/2_ → ^2^F_7/2_ transition of the Yb^3+^ ion.

To investigate the nature of this ligand-centered emission, photoluminescence decay measurements were recorded (Figure S11). The lifetime values obtained for the three emission components discussed above (<0.2 ns for λ_em_ = 370 nm, 0.45 ns for λ_em_ = 415 nm and 3.0 ns for λ_em_ = 630 nm) are consistent with fluorescence-based deactivation processes, as expected from the photophysical behavior reported for similar related compounds.^41,42^

Although the Yb^3+^ ion possesses a relatively simple electronic structure, the spectroscopic interpretation of its metal complexes and the reliable reconstruction of their energy level schemes are often challenging due to the significant contribution of vibronic side-bands typically observed in this type of systems.^43^ In order to support the assignment of the observed transitions, *ab initio* calculations were performed in combination with low-temperature spectroscopic measurements.

The low-temperature emission spectrum revealed the Stark splitting associated with the ^2^F_5/2_ → ^2^F_7/2_ transition, allowing the identification of the different Kramers doublets of the Yb^3+^ ion (Figure 6). The analysis of the experimental spectrum together with the theoretical calculations enabled the assignment of the observed transitions and the reconstruction of the corresponding energy level scheme of the investigated system (*vide infra*).

Since Yb^3+^ is a Kramers ion, the maximum crystal-field splitting of each multiplet should generate (2*J+1)/2* Kramers doublets. Therefore, for the ^2^F_7/2_ ground state, a maximum of four KDs is expected. However, in the case of complex **1**, the experimental spectrum exhibits at least six distinguishable components, exceeding the number expected. These two additional transitions seem to be hot bands, originated from the second and/or the third Stark sublevels of the excited ^2^F_5/2_ state.^44^ The deconvolution of the spectra allowed the determination of the relative energies of each KD (Figure S12 and Table S5), revealing an energy gap of 496.54 cm^–1^ between the first two KDs of the ^2^F_7/2_ ground state for complex **1**, in close agreement with *ab initio* calculations (*vide infra*).

In contrast, the experimental spectrum of complex **2·**CH_3_CH_2_OH·H_2_O in the NIR region can be satisfactorily reproduced by deconvolution into four Gaussian components, as expected for the four Kramers doublets associated with the ^2^F_7/2_ ground state. The analysis reveals an energy gap of 353.92 cm^–1^, significantly smaller than that observed for **1**, highlighting the influence of the auxiliary ligands on the crystal-field splitting.

### *Ab Initio* Calculations

To obtain a more detailed understanding of the magnetic anisotropy exhibited by the complexes and to support the experimental findings, multireference quantum-chemical calculations were performed using the complete active space self-consistent field (CASSCF) approach, second-order N-electron valence state perturbation theory (NEVPT2), and its quasi-degenerate formulation (QD-NEVPT2). These calculations employed the molecular structures determined by single-crystal X-ray diffraction as input geometries (see the Experimental section for computational details). At the CASSCF level, the splitting of the ground-state multiplet of Yb^3+^ (Table S6) leads to the best agreement with the energy gaps extracted from the luminescence measurements. The inclusion of dynamic correlation effects through the QD-NEVPT2 approach does not improve the agreement with the experimental data (see SI).

For the Yb^3+^ ion, the ^2^F_7/2_ ground level splits into four Kramers doublets (KDs). For **1**, the KDs span an energy range up to 666.51 cm^–1^, whereas for **2** the splitting extends up to 775.22 cm^–1^ (Table S6). Despite this difference in the overall splitting, the energy of the first excited Kramers doublet (KD2) is remarkably similar in both complexes.

For the ground Kramers doublet, the *g*_*z*_ component is much larger than the others, which indicates a mainly axial magnetic anisotropy (Figure 7). For both complexes, the principal magnetic axes associated with the ground Kramers doublets are oriented nearly perpendicular to the plane defined by the *N*_3_ *O*_2_ donor set (Figure 7).

**Figure 7:**
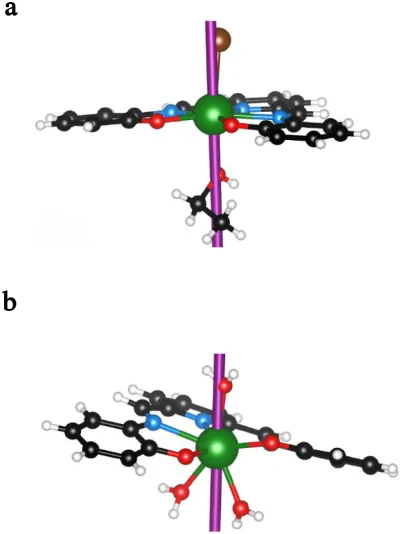
Orientation of the main magnetic axis (associated with the largest g value, *g_z_*) of the ground Kramers doublet on Yb^3+^ for: (a) **1**; (b) **2·**CH_3_CH_2_OH·H_2_O (Figures generated with VESTA).^45^

However, *g*_*x*_ and *g*_*y*_ components are nonzero (Tables S7 and S8). The occurrence of transverse terms indicates *M*_*J*_ state mixing and facilitates quantum tunneling of magnetization, allowing an effective pathway for magnetic relaxation.

To confirm this, we analyzed the magnetic transition strengths between the Kramers doublets (Figure 8) of the ground level. The relatively large values between the components of the ground Kramers doublet indicate efficient QTM at zero field. The experimental data show that applying a *dc* magnetic field partially suppresses QTM (Figure 4), thereby enabling the observation of slow magnetization relaxation.

**Figure 8:**
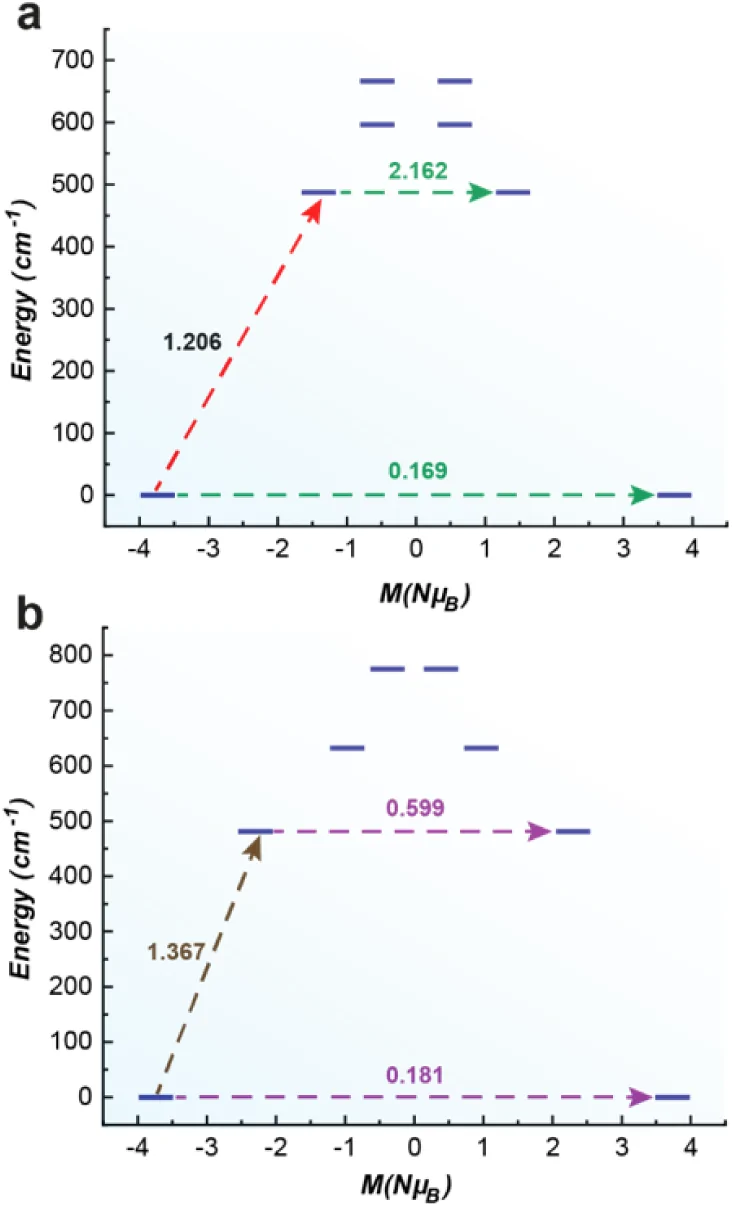
Estimation of the probability using CASSCF calculations for different relaxation mechanisms of the spin between the two low energy KDs (blue lines) for: a) **1**; b) **2**: QTM and TA-QTM (green and purple arrows); spin-phonon relaxation pathways (red and brown arrows). The values next to the arrows correspond to the average square magnitude of the matrix elements of the transition magnetic moment components $\frac{1}{3}\left({|\mu_{x}|}^{2}+{|\mu_{y}|}^{2}+{|\mu_{z}|}^{2}\right)$ .^46^ When this value is above 0.1 an efficient spin relaxation mechanism is expected.

In both cases, the first excited Kramers doublet lies at *ca*. 490 cm^–1^ (Figure 8), representing the lower bound of the energy barrier relevant for a possible Orbach pathway, very far away from the values calculated from *ac* measurements (14.9 and 22.1 cm^–1^ for **1** and **2·**CH_3_CH_2_OH·H_2_O, respectively). Accordingly, *ab initio* calculations clarify the pathway of magnetic relaxation: (i) efficient QTM prevents the experimental observation of SIM behavior at zero field, and (ii) the presence of an Orbach process, considering the lower barrier heights obtained from fitting the temperature-dependent relaxation, can be discarded in favor of a combination of Raman and QTM processes.

Finally, the calculations show that replacing the auxiliary bromide and ethanol ligands by coordinated water has only a minor effect on the magnetic properties. In particular, the energy of the first excited Kramers doublet and the calculated QTM probabilities remain essentially unchanged, indicating that the auxiliary ligands exert only a limited influence on the magnetic relaxation dynamics.

These *ab initio* calculated energy barriers are also in quite good agreement with the spectroscopic results. Thus, for **1** the energy barriers are very similar (487.26 and 496.54 cm^–1^) while for **2** the slight discrepancy between the barriers calculated for both methods (481.12 and 353.92 cm^–1^) is not unprecedented,^8,12^ and may arise from solvent and crystal-packing effects not fully accounted for in the CASSCF treatment. For instance, the extensive hydrogen-bonding network involving coordinated and lattice solvent molecules in **2·**CH_3_CH_2_OH·H_2_O, which is not possible in **1**, can affect the crystal field and consequently the magnetic properties. The disregard of its explicit consideration in the computational model, which was not attempted in the current study, might explain the discrepancies between the experimental and theoretical results.

Assignment of the experimental emission bands was also carried out using the crystal-field splitting of the Yb^3+^ ions derived from CASSCF calculations. Figure 9 presents the experimental emission spectra and simplified energy-level diagrams. In Yb^3+^, the relevant transition corresponds to ^2^F_5/2_ → ^2^F_7/2_ where the excited level (denoted Y) splits into three Kramers doublets (Y1–Y3) and the ground level (denoted Z) into four (Z1–Z4).

**Figure 9:**
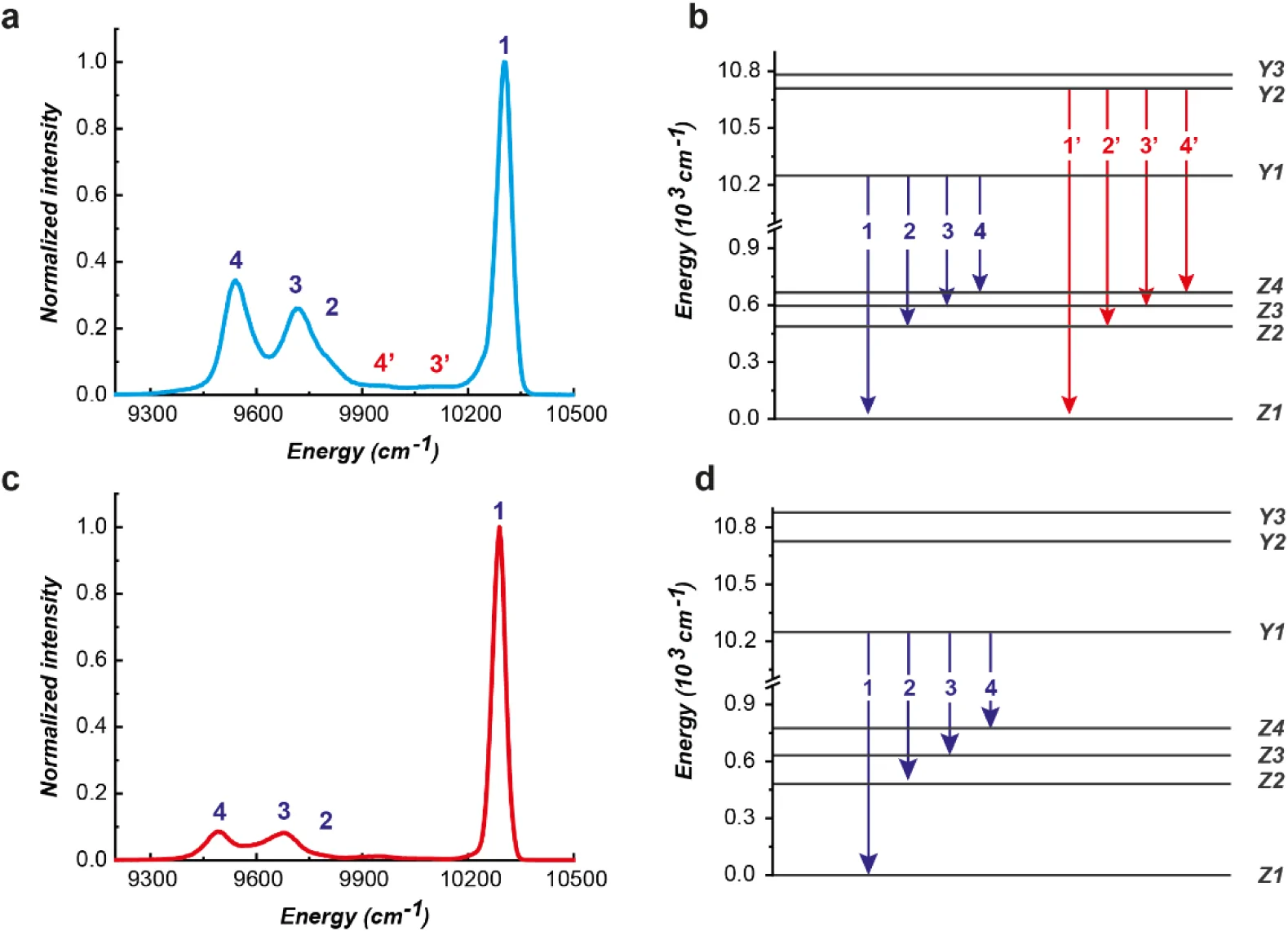
Emission spectra at 15 K of **1** (a) and **2** (c), and scheme of the main emission transitions for **1** (b) and for **2** (d).

The most intense band (1) is assigned to the Y1 → Z1 transition. The subsequent bands (2–4), observed at lower energies, are attributed to transitions from the lowest excited Stark level Y1 to higher-lying ground-state components, namely Y1 → Z2, Y1 → Z3, and Y1 → Z4, respectively. In addition, weaker features marked as 3′ and 4′ are assigned to hot-band transitions for **1**, originating from thermally populated higher-energy excited sublevel Y2, involving transitions such as Y2 → Z3 and Y2 → Z4.

Accordingly, the calculations indicate that replacing the bromide and ethanol ligands in **1** with water ligands in **2** modifies the Stark splitting of the excited ^2^ *F*_5/2_ multiplet. As a result, the thermal population of the higher-energy excited sublevels is reduced in **2** relative to **1**, explaining the absence of hot-band contributions for **2**.

### Luminescence Thermometry

The thermometric behavior of **1** and **2·**CH_3_CH_2_OH·H_2_O was investigated by recording and analyzing the emission spectra in the interval 15–310 K (Figures 10a and S13–S15).

**Figure 10:**
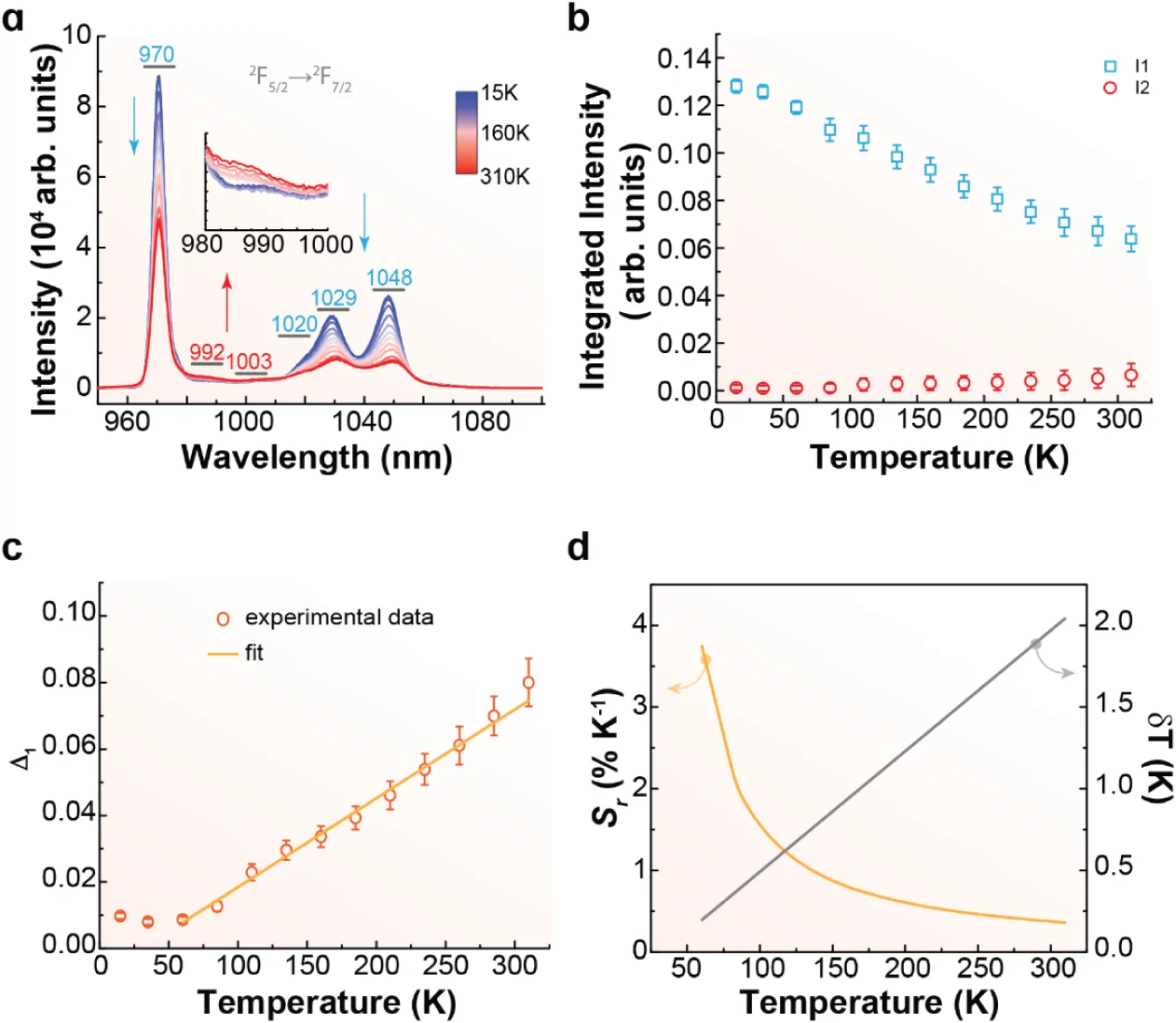
(a) Enlarged view of the Yb^3+^ temperature-dependent ^2^F_5/2_ → ^2^F_7/2_ transition in the NIR emission spectra of complex **1** (b) temperature evolution of the integrated intensities *I*_1_ and *I*_2_. (c) Temperature dependence of the thermometric parameter Δ_1_. The solid line represents the best fit of the experimental data to a linear function (fit details in Table S9). (d) Temperature dependence of the relative sensitivity (*S_r_*) and temperature uncertainty (*δT*) associated with Δ_1_.

In the case of complex **1**, and considering the results from *ab initio* calculations, the emission spectrum was deconvoluted as previously discussed. This deconvolution made it possible to analyze the thermal evolution of each Stark component individually (Figure S14). The three main peaks in the Yb^3+^ emission spectrum (970, 1029, and 1048 nm or 10310, 9718, and 9542 cm^–1^) were assigned to the Y1 → Z1, Y1 → Z3, and Y1 → Z4 transitions, respectively, while the shoulder observed at 1020 nm (9803 cm^–1^) corresponds to the Y1 → Z2 transition (see Figure 9). Transitions originating from higher excited states (Y2) exhibit significantly lower intensities due to their lower population and are grouped around the bands centered at 992 nm (10081 cm^–1^) and 1003 nm (9970 cm^–1^), in agreement with the experimental results. Furthermore, the calculations confirm the assignment of the Y2 → Z3 and Y2 → Z4 transitions for these bands.

For the transitions originating from the Y1 level, the emission intensities decrease with increasing temperature, whereas the bands associated with the Y2 → Z3 and Y2 → Z4 transitions show a slight thermal increase, consistent with their assignment as hot bands, although an additional vibronic contribution, as previously reported for Yb^3+^ systems, cannot be excluded.^43,47^ Considering this thermal behavior, a thermometric parameter (Δ_1_) was defined as the ratio between the summed integrated areas of the two deconvoluted Gaussian peaks assigned to the hot bands (*I*_2_: 992 and 1003 nm) and the summed integrated areas of the four deconvoluted Gaussian peaks originating from the Y1 level (*I*_1_: 1048, 1029, 1020, and 970 nm). As shown in Figure 10b, while *I*_2_ increases with temperature, *I*_1_ progressively decreases.

The thermal dependence of Δ_1_ (Figure 10c) shows almost constant behavior up to 60 K, followed by a linear increase at higher temperatures. Although the increase of I_2_ is consistent with thermal population of the Y_2_ level, the ln (Δ_1_) versus 1/*T* plot (Figure S13) does not follow a single Boltzmann dependence over the whole temperature range, suggesting that additional contributions, such as vibronic transitions, may also be present. Therefore, the temperature dependence could be described using an empirical linear function (Table S9).^47^ The performance of this ratiometric thermometric approach was evaluated by calculating the relative sensitivity (*S_r_*) within the operational range of 60–310 K. The system exhibits a maximum relative sensitivity of *S*_*r*_ = 3.7% K^–1^ at 60 K and a minimum temperature uncertainty (*δT*) of 0.2 K at the same temperature. To the best of our knowledge, this represents the highest relative sensitivity reported so far for a mononuclear Yb^3+^ complex displaying field-induced single-molecule magnet behavior (Table S10), while remaining competitive with those achieved by recently reported luminescent Yb^3+^ complexes lacking magnetic functionality.^47^

Moreover, the thermometric analysis has been completed with the measurement of variable-temperature NIR emission lifetimes, which show the expected trend with temperature (i.e., lifetime value decreases as temperature is increased from 10 to 310 K, dropping from 1.72 to 1.22 μs for **1** and from 1.43 to 1.01 μs for **2**) associated with the thermal vibrational quenching usually observed for coordination complexes (Figures S16–S17).

In the case of complex **2**·CH_3_CH_2_OH·H_2_O, no hot bands were observed in the emission spectra, which show less intense emission bands (Figure S15a). This lower intensity is expected, as replacing the auxiliary bromide and ethanol ligands in **1** with three coordinated water molecules in **2** introduce additional high-energy O–H oscillators that promote non-radiative multiphonon relaxation as supported by recorded lifetimes (Figure S10), thereby quenching the Yb^3+^ emission, making **1** intrinsically more favorable for efficient luminescence.^48^ In spite of this, the relative intensity between the Stark components of the Yb^3+^ emission varies with temperature, allowing the definition of an alternative thermometric parameter (Δ_2_) based on the ratio between two spectral regions. In this case, Δ_2_ was defined as *I*_1_/*I*_2_, where *I*_1_ and *I*_2_ correspond to the integrated intensities in the 950–980 and 980–1000 nm spectral regions, respectively (Figure S15a). As shown in Figure S15b, *I*_1_ progressively decreases with increasing temperature, whereas *I*_2_ exhibits a slight thermal increase. Consequently, the Δ_2_ values display an approximately linear temperature dependence over the whole studied temperature range (Figure S15c). The thermal dependence of Δ_2_ could be satisfactorily described using an empirical linear function (Table S9). The system exhibits a maximum relative sensitivity of *S_r_* = 0.4% K^–1^ at 310 K and a *δT* of 0.24 K at the same temperature (Figure S15d). These values are significantly lower than those observed for **1** and place **2**·CH_3_CH_2_OH·H_2_O among the least sensitive Yb^3+^ field-induced single-molecule magnets reported to date (Table S10). Thus, as previously stated, this marked contrast underscores the crucial role of the auxiliary monodentate ligands in modulating the thermometric response, demonstrating that relatively subtle changes in the coordination sphere can strongly influence luminescence-based thermal sensing while exerting only a minor influence on the magnetic properties.

The thermometric analysis has been completed with the measurement of variable-temperature NIR emission lifetimes, which show the expected trend with temperature (i.e., lifetime value decrease as temperature is increased from 10 to 310 K, dropping from 1.72 to 1.22 μs for **1** and from 1.43 to 1.01 μs for **2**) associated with the thermal vibrational quenching usually observed for coordination complexes (Figures S16–S17).

## Conclusions

The new Yb^3+^ complexes **1** and **2**·CH_3_CH_2_OH·H_2_O, derived from the *N*_3_ *O*_2_ Schiff-base H_2_L, have been synthesized and structurally characterized, revealing markedly different coordination environments induced by the auxiliary ligands. Despite these structural differences, both compounds exhibit comparable field-induced single-ion magnet behavior, with magnetic relaxation governed by QTM and Raman processes. In contrast, their luminescent thermometric performances differ dramatically, demonstrating that the ancillary donors exert a much stronger influence on the optical properties than on the magnetic dynamics. Accordingly, replacement of bromide and ethanol donors by water ligands seems to enhance non-radiative multiphonon relaxation through O–H vibrations, making **1** intrinsically more favorable for efficient Yb^3+^ luminescence. Hence, **1** displays outstanding thermometric performance, achieving a maximum relative sensitivity of 3.7% K^–1^ and a minimum temperature uncertainty of 0.2 K, representing the highest sensitivity reported to date for a mononuclear Yb^3+^ field-induced single-ion magnet. *Ab initio* calculations are in excellent agreement with the experimental magnetic and spectroscopic data, confirming that coligand substitution produces only a minor effect on the electronic structure governing the magnetic relaxation pathways whereas it significantly alters the thermal population of the Yb^3+^ energy levels involved in the emission processes. Overall, this work expands the still limited family of Yb^3+^ single-ion magnets with luminescent thermometric behavior and demonstrates that auxiliary ligand modification is an effective strategy to optimize thermometric performance. More importantly, these results reveal that magnetic relaxation and luminescent thermometry can be, to a significant extent, independently tuned in lanthanoid systems. This provides simple design guidelines for developing multifunctional lanthanoid-based molecular materials capable of combining information storage with high-performance optical temperature sensing and readout.

## Supplementary Material



## Notes

Additional figures, X-ray tables, and SHAPE calculations (PDF)

## References

[1] Rinehart J. D., Long J. R. (2011). Exploiting single-ion anisotropy in the design of f-element
single-molecule magnets. Chem. Sci..

[2] Zabala-Lekuona A., Seco J. M., Colacio E. (2021). Single-molecule magnets: from Mn12-ac
to dysprosium metallocenes, a travel in time. Coord. Chem. Rev..

[3] Emerson-King J., Gransbury G. K., Atkinson B. E., Blackmore W. J. A., Whitehead G. F. S., Chilton N. F., Mills D. P. (2025). Soft magnetic
hysteresis in a dysprosium amide–alkene complex up to 100 K. Nature.

[4] Corredoira-Vázquez J., González-Barreira C., Fondo M., García-Deibe A.
M., Sanmartín-Matalobos J., Gómez-Coca S., Ruiz E., Brites C. D. S., Carlos L. D. (2025). An air-stable
high-performance
single-molecule magnet operating as a luminescent thermometer below
its blocking temperature. Inorg. Chem. Front..

[5] Xu W.-J., Luo Q.-C., Li Z.-H., Zhai Y.-Q., Zheng Y.-Z. (2024). Bis-alkoxide
dysprosium­(III) crown ether complexes exhibit tunable air stability
and record energy barrier. Adv. Sci..

[6] Nielsen V. R. M., Sørensen T. J. (2025). Electronic
energy levels and optical
spectra of trivalent lanthanide ions in water – Ce­(III), Pr­(III),
Nd­(III), Sm­(III), Eu­(III), Gd­(III), Tb­(III), Dy­(III), Ho­(III), Er­(III),
Tm­(III) and Yb­(III). Nat. Commun..

[7] Pushkarev A. P., Balashova T. V., Kukinov A. A., Arsenyev M. V., Yablonskiy A. N., Kryzhkov D. I., Andreev B. A., Rumyantcev R. V., Fukin G. K., Bochkarev M. N. (2017). Sensitization of NIR luminescence
of Yb^3+^ by Zn^2+^ chromophores in heterometallic
complexes with a bridging Schiff-base ligand. Dalton Trans..

[8] Brunet G., Marin R., Monk M.-J., Resch-Genger U., Gálico D. A., Sigoli F. A., Suturina E. A., Hemmer E., Murugesu M. (2019). Exploring the dual functionality
of an ytterbium complex
for luminescence thermometry and slow magnetic relaxation. Chem. Sci..

[9] Fondo M., Corredoira-Vázquez J., García-Deibe A. M., Sanmartín-Matalobos J., Amoza M., Botas A. M. P., Ferreira R. A. S., Carlos L. D., Colacio E. (2020). Field-induced
slow magnetic relaxation and luminescence thermometry in a mononuclear
ytterbium complex. Inorg. Chem. Front..

[10] Kumar K., Stefanczyk O., Nakabayashi K., Imoto K., Oki Y., Ohkoshi S.-I. (2022). Detection
of Sub-terahertz Raman response and nonlinear
optical effects for luminescent Yb­(III) complexes. Adv. Opt. Mater..

[11] Wang J., Zakrzewski J. J., Heczko M., Zychowicz M., Nakagawa K., Nakabayashi K., Sieklucka B., Chorazy S., Ohkoshi S.-I. (2020). Proton conductive luminescent thermometer
based on near-infrared emissive {YbCo2} molecular nanomagnets. J. Am. Chem. Soc..

[12] Karachousos-Spiliotakopoulos K., Tangoulis V., Panagiotou N., Tasiopoulos A., Moreno-Pineda E., Wernsdorfer W., Schulze M., Botas A. M. P., Carlos L. D. (2022). Luminescence thermometry and field induced slow magnetic
relaxation based on a near infrared emissive heterometallic complex. Dalton Trans..

[13] Xin Y., Kumar K., Zakrzewski J. J., Chorazy S., Nakabayashi K., Stefanczyk O., Ohkoshi S.-I. (2023). Tuning of ratiometric optical thermometry
in the family of magnetic {Nd_1–x_
^III^Yb^IIIx^[Cu_2_I­(CN)_5_]} frameworks showing the
large SHG response. J. Phys. Chem. C.

[14] González A., Gómez E., Cortés-Lozada A., Hernández S., Ramírez-Apan T., Nieto-Camacho A. (2009). Heptacoordinate tin­(IV) compounds
derived from pyridine Schiff bases: synthesis, characterization, in
vitro cytotoxicity, anti-inflammatory and antioxidant activity. Chem. Pharm. Bull..

[15] Corredoira-Vázquez J., Oreiro-Martínez P., García-Deibe A. M., Sanmartín-Matalobos J., Fondo M. (2023). A Dy^III^ complex
of a pentadentate Schiff base with field-induced single-ion magnet
behaviour. Magnetochemistry.

[16] Sheldrick, G. M. SADABS, Area-Detector Absorption Correction, Siemens Industrial Automation, Inc. Madison, WI; 2001.

[17] Sheldrick G. M. (2015). SHELXT
- Integrated space-group and crystal-structure determination. Acta Crystallogr.

[18] Sheldrick G. (2015). Crystal structure
refinement with SHELXL. Acta Crystallogr., Sect.
C.

[19] Mooney J., Kambhampati P. (2013). Get the basics right: Jacobian conversion
of wavelength
and energy scales for quantitative analysis of emission spectra. J. Phys. Chem. Lett..

[20] Mooney J., Kambhampati P. (2014). Correction
to “Get the basics right: Jacobian
conversion of wavelength and energy scales for quantitative analysis
of emission spectra”. J. Phys. Chem.
Lett..

[21] Mooney J., Krause M. M., Saari J. I., Kambhampati P. (2013). A microscopic
picture of surface charge trapping in semiconductor nanocrystals. J. Chem. Phys..

[22] Krause M. M., Mooney J., Kambhampati P. (2013). Chemical and thermodynamic control
of the surface of semiconductor nanocrystals for designer white light
emitters. ACS Nano.

[23] Myers A. B. (1994). Relating
absorption, emission, and resonance Raman spectra with electron transfer
rates in photoinduced charge transfer systems: promises and pitfalls. Chem. Phys..

[24] Brites C. D. S., Balabhadra S., Carlos L. D. (2019). Lanthanide-based
thermometers: at
the cutting-edge of luminescence thermometry. Adv. Opt. Mater..

[25] Brites C. D. S., Lima P. P., Silva N. J. O., Millán A., Amaral V. S., Palacio F., Carlos L. D. (2011). Lanthanide-based
luminescent molecular thermometers. New J. Chem..

[26] Ramos T. J.
S., Longo R. L., Brites C. D. S., Ferreira R. A. S., Malta O. L., Carlos L. D. (2023). Exploring
the intra-4f and the bright white light upconversion
emissions of Gd_2_O_3_: Yb^3+^,Er^3+^-based materials for thermometry. Nanoscale.

[27] Neese F. (2025). Software update:
the ORCA program system—Version 6.0. WIREs Comput. Mol. Sci.

[28] Reiher M. (2006). Douglas–Kroll–Hess
Theory: a relativistic electrons-only theory for chemistry. Theor. Chem. Acc..

[29] Malmqvist P.-Å., Roos B. O. (1989). The CASSCF state
interaction method. Chem. Phys. Lett..

[30] Angeli C., Cimiraglia R., Malrieu J.-P. (2001). N-electron valence state perturbation
theory: a fast implementation of the strongly contracted variant. Chem. Phys. Lett..

[31] Angeli C., Borini S., Cestari M., Cimiraglia R. (2004). A quasidegenerate
formulation of the second order n-electron valence state perturbation
theory approach. J. Chem. Phys..

[32] Heß B.
A., Marian C. M., Wahlgren U., Gropen O. (1996). A mean-field spin-orbit
method applicable to correlated wavefunctions. Chem. Phys. Lett..

[33] Roos B. O., Lindh R., Malmqvist P.-Å., Veryazov V., Widmark P.-O., Borin A. C. (2008). New relativistic
atomic natural orbital basis sets
for lanthanide atoms with applications to the Ce diatom and LuF_3_. J. Phys. Chem. A.

[34] Stoychev G. L., Auer A. A., Neese F. (2017). Automatic
generation of auxiliary
basis sets. J. Chem. Theory Comput..

[35] Neese F. (2003). An improvement
of the resolution of the identity approximation for the formation
of the Coulomb matrix. J. Comput. Chem..

[36] Fondo M., Corredoira-Vázquez J., García-Deibe A. M., Gómez-Coca S., Ruiz E., Sanmartín-Matalobos J. (2020). Dysprosium-based
complexes with a flat pentadentate donor: a magnetic and ab initio
study. Dalton Trans..

[37] a Llunell, M. ; Casanova, D. ; Cirera, J. ; Alemany, P. ; Alvarez, S. , SHAPE, version 2.1, Universitat de Barcelona, Barcelona, Spain, 2013.

[38] Singh A., Shrivastava K. N. (1979). Optical-acoustic two-phonon relaxation in spin systems. Phys. Status Solidi B.

[39] Gavrikov A. V., Efimov N. N., Dobrokhotova Z. V., Ilyukhin A. B., Vasilyev P. N., Novotortsev V. M. (2017). Novel mononuclear
Ln complexes with pyrazine-2-carboxylate
and acetylacetonate co-ligands: remarkable single molecule magnet
behavior of a Yb derivative. Dalton Trans..

[40] Bodman S.
E., Butler S. J. (2021). Advances
in anion binding and sensing using luminescent
lanthanide complexes. Chem. Sci..

[41] Chiang H.-W., Su Y.-T., Wu J.-Y. (2013). Ligand
dissociation/recoordination
in fluorescent ionic zinc–salicylideneimine compounds: synthesis,
characterization, photophysical properties, and 1H NMR studies. Dalton Trans.

[42] Yao Y., Yin H.-Y., Ning Y., Wang J., Meng Y.-S., Huang X., Zhang W., Kang L., Zhang J.-L. (2019). Strong
fluorescent lanthanide salen complexes: photophysical properties,
excited-state dynamics, and bioimaging. Inorg.
Chem..

[43] Paul-Henri H., Romain G., Bruno V., Elisabeth A.-F., Daniel V. (2001). Spectroscopic and crystal-field analysis of new Yb-doped
laser materials. J. Phys.: Condens. Matter.

[44] Yi X., Bernot K., Le Corre V., Calvez G., Pointillart F., Cador O., Le Guennic B., Jung J., Maury O., Placide V. (2014). Unraveling
the Crystal Structure of Lanthanide–Murexide
Complexes: Use of an Ancient Complexometry Indicator as a Near-Infrared-Emitting
Single-Ion Magnet. Chem. - Eur. J.

[45] Momma K., Izumi F. (2011). VESTA 3 for three-dimensional
visualization of crystal, volumetric
and morphology data. J. Appl. Crystallogr..

[46] Chilton N. F. (2025). Ab initio
electronic structure calculations of lanthanide single-molecule magnets;
a practical guide. Chem. Soc. Rev..

[47] Gil Y., Corredoira-Vázquez J., Costa de Santana R., Vega A., Carneiro Neto A. N., Aravena D., Spodine E., Brites C. D. S., Carlos L. D., Fuentealba P. (2026). Triple-readout
luminescence thermometry in a heterodinuclear Yb–Er complex
and performance comparison with the homodinuclear analogues. J. Mater. Chem. C.

[48] Parker D., Fradgley J. D., Wong K.-L. (2021). The design
of responsive luminescent
lanthanide probes and sensors. Chem. Soc. Rev..

