# Supplementary material for: Tuning Luminescent Thermometry in Ytterbium N3O2 Field-Induced Single-Molecule Magnets via Auxiliary Ligands
Source: Inorg Chem. 2026 Sep 16;65(38):22441–52. doi: 10.1021/acs.inorgchem.6c03015 (PMC13625969; doi:10.1021/acs.inorgchem.6c03015)
Supplement: Supplementary file 1 [file ic6c03015_si_001.pdf]

SUPPLEMMENTARY INFORMATION

Tuning Luminescent Thermometry in Ytterbium N_3O_2 Field-induced Single Molecule Magnets *via* Auxiliary Ligands

Paula Oreiro-Martínez,[‡,⊥] *Julio Corredoira-Vázquez,*[‡,ε,*] *Ana M. García-Deibe,*[‡] *Javier Cepeda,*[§] *Enrique Colacio,*[$] *Federico Totti,*[⊥] *Jakob K. Staab*[⊥] *and Matilde Fondo,*[‡,ε,*]

[‡] Departamento de Química Inorgánica, Facultade de Química, Universidade de Santiago de Compostela, 15782 Santiago de Compostela, Spain. E-mail: matilde.fondo@usc.es; julio.corredoira.vazquez@usc.es

[⊥] Department of Chemistry "U. Schiff", University of Florence, 50019, Sesto Fiorentino Italy

[ε] Institute of Materials (iMATUS), Universidade de Santiago de Compostela, 15782 Santiago de Compostela, Spain

[§] Departamento de Química Aplicada, Facultad de Química, Euskal Herriko Unibertsitatea (EHU), 20018 Donostia-San Sebastián, Spain

[$] Departamento de Química Inorgánica, Facultad de Ciencias, Universidad de Granada, Avda Fuentenueva s/n, 18071 Granada, Spain

1. STRUCTURAL CHARACTERIZATION S5

Table S1. Crystal data and structure refinement for **1**·CH_3OH and **2**·CH_3CH_2OH·H_2O. S5

Table S2. Main bond distances (Å) and angles (°) for **1**·CH_3OH, including H-bond. S6

Table S3. Main bond distances (Å) and angles (°) for **2**·CH_3CH_2OH·H_2O, including H-bond. S6

Table S4. SHAPE v2.1. Continuous Shape Measures Calculation (c) 2013 Electronic Structure Group, Universitat de Barcelona. S7

Figure S1. IR spectra of **1** (up) and **2**·CH_3CH_2OH·H_2O (bottom). S8

Figure S2. Balls and sticks diagram for **1**·CH_3OH illustrating the zigzag chain structure stabilized by intermolecular hydrogen bonds. S9

Figure S3. Balls and sticks diagram for **2**·CH_3CH_2OH·H_2O illustrating the zigzag chain structure stabilized by intermolecular hydrogen bonds. S9

Figure S4. Comparative powder X-ray diffractograms for **2**·CH_3CH_2OH·H_2O (black) and the simulation from single X-ray diffraction data (red). S9

2. MAGNETIC CHARACTERIZATION S10

Figure S5. Dependence of the relaxation time with the field for 1 at 3 K. The solid line is a guide for the eyes. S10

Figure S6. Cole Cole plots for **1** (left) and **2**·CH_3CH_2OH·H_2O (right) in Hdc = 1500 Oe. S10

Figure S7. Temperature dependence of χ''_M for **1** (left) and **2**·CH_3CH_2OH·H_2O (right) in H_{dc} = 1500 Oe at different frequencies. The solid lines are guides for the eyes. S10

3. SPECTROSCOPIC CHARACTERIZATION S11

Figure S8. Room-temperature emission spectra of complexes **1** and **2**·CH_3CH_2OH·H_2O recorded under excitation at 325 nm. S11

Figure S9. Solid state UV-Vis absorption spectra for compounds **1** and **2**·CH_3CH_2OH·H_2O. Note that tail fitting was employed to estimate the lifetimes. S11

Figure S10. Comparative analysis of the decay curves of the Yb-centered NIR emission under UV illumination (λex = 325 nm) for compounds **1** and **2**· CH_3CH_2OH·H_2O. Note that tail fitting was employed to estimate the lifetimes. S11

Figure S11. Decay curves of the ligand-centered emission components taken at room temperature under UV LED illumination (λex = 340 nm) on a powdered polycrystalline sample of **1**. Note that reconvolution fitting was employed to estimate the lifetimes owing to the short life of the processes, which are close to the pulse of the LED. S12

Figure S12. Temperature-dependent emission spectra and spectral deconvolution of compounds **1** and **2**·$CH_3CH_2OH·H_2O$ recorded at 15 and 210 K upon excitation at 325 nm**.** Panels **(a)** and **(b)** correspond to compound **1** at 15 and 210 K, respectively, whereas panels **(c)** and **(d)** show compound **2**·$CH_3CH_2OH·H_2O$ at 15 and 210 K**,** respectively. The blue circles represent the experimental data, while the **red line** corresponds to the overall fit obtained using multiple Gaussian functions S12

Table S5. Energy of the Kramers doublets (KDs) obtained from spectroscopic measurements of complexes **1** and **2**·$CH_3CH_2OH·H_2O$ S13

4. Ab Initio CALCULATIONS S13

Table S6. Energies of the Kramers doublets within the ground state of **1** and **2**. S13

Table S7. Components of the g-tensor and tilting angle (θ) of the eigenvector associated to the g_z component of the corresponding excited state KD with respect to the ground state KD for **1**, derived from CASSCF calculations. S13

Table S8. Components of the g-tensor and tilting angle (θ) of the eigenvector associated to the g_z component of the corresponding excited state KD with respect to the ground state KD for **2** derived from CASSCF calculations. S13

5. LUMINESCENCE THERMOMETRY S14

Figure S13. Temperature-dependent of the integrated Stark component areas for the Yb^{3+} $^2F_{5/2}\rightarrow^2F_{7/2}$ transition in **1**. S14

Figure S14. (a) Temperature-dependent Yb^{3+} $^2F_{5/2}\rightarrow^2F_{7/2}$ transition in the NIR emission spectra of complex **2**·$CH_3CH_2OH·H_2O$ in the 15-310 K temperature range. (b) Temperature evolution of the integrated intensities in the 950-980 and 980-1000 nm spectral regions (c) Temperature dependence of the thermometric parameter Δ_2. The solid line represents the best fit of the experimental data to a linear function (fit details in Table S9). (d) Temperature dependence of the relative sensitivity (S_r) and temperature uncertainty (δT) associated with Δ_2 S14

Table S9. Summary of the fitting parameters for the plots of complexes 1 and **2**·$CH_3CH_2OH·H_2O$ S15

Figure S15. Temperature dependence of ln(Δ_1) as a function of inverse temperature (1/*T*). S15

Figure S16. Variable-temperature decay curves of the Yb-centered NIR emission under UV illumination (λex = 325 nm) on a powdered polycrystalline sample of **1**. Note that tail fitting was employed to estimate the lifetimes. S16

Figure S17. Variable-temperature decay curves of the Yb-centered NIR emission under UV illumination (λex = 325 nm) on a powdered polycrystalline sample of **2**·CH_3CH_2OH·H_2O. Note that tail fitting was employed to estimate the lifetimes. S17
Table S10. Excitation wavelength (λ), temperature range (ΔT), maximum relative sensitivity (S_m), and corresponding temperature (T_m) of the Yb^{3+}-based thermometers with slow relaxation of the magnetization reported so far. S18
REFERENCES S19

1. STRUCTURAL CHARACTERIZATION

Table S1. Crystal data and structure refinement for **1**·CH_3OH and **2**·$CH_3CH_2OH·H_2O$.

	**1**·CH_3OH	**2**·$CH_3CH_2OH·H_2O$
Empirical formula	$C_{22}H_{23}BrN_3O_4Yb$	$C_{21}H_{27}IN_3O_7Yb$
Molecular weight	646.38	733.39
Crystal system	Monoclinic	Monoclinic
Space group	*P* 21/C	*P* 21/n
Wavelength (Å)	0.71073	0.71073
Crystal size (mm^3)	0.20 x 0.08 x 0.04	0.16 x 0.11 x 0.04
Colour, shape	Red, needle	Red, plate
T (K)	293	100
a (Å)	8.2804(16)	13.5439(13)
b (Å)	14.338(3)	8.7608(8)
c (Å)	18.779(3)	20.984(2)
α (º)	90	90
β (º)	99.840(6)	105.497(4)
γ (º)	90	90
Volume (Å^3)	2196.7(7)	2399.38(4)
Z	4	4
Absorpt. coef. (mm^{-1})	6.110	5.231
Reflections collected	131481	71708
Independent reflections	6432 [R_{int}= 0.0686]	7322 [R_{int}= 0.0561]
Data / restrains / param.	6432 / 0 / 287	7322 / 0 / 381
Final R indices [I>2σ(I)]	R_1 = 0.0180; wR_2 = 0.0383	R_1 = 0.0197; wR_2 = 0.0429
R indices (all data)	R_1 = 0.0232; wR_2 = 0.0397	R_1 = 0.0251; wR_2 = 0.0447

Table S2. Main bond distances (Å) and angles (°) for **1**·CH_3OH, including H-bond.

Yb1-O2	2.1654(14)	O1S-Yb1-Br1	165.29(4)	
Yb1-O1	2.2075(15)	N2-Yb1-Br1	85.48(4)	
Yb1-O1S	2.2927(16)	O1S-Yb1-N2	79.90(6)	
Yb1-N2	2.4147(17)	N2-Yb1-N3	65.57(6)	
Yb1-N1	2.4304(17)	N1-Yb1-N3	131.43(6)	
Yb1-N3	2.4415(17)	O2-Yb1-N2	135.48(6)	
Yb1-Br1	2.7407(5)	O2-Yb1-O1	88.04(5)	
D-H...A	d(D-H)	d(H...A)	d(D...A)	<(DHA)
O1S-H1S...O1	0.80	1.80	2.592(2)	179.3
O2S-H2S...O1[#3]	0.82	1.89	2.708(2)	178.8

[#3] -x+1,y+1/2,-z+3/2

Table S3. Main bond distances (Å) and angles (°) for **2**·CH_3CH_2OH·H_2O, including H-bond.

Yb1-O2	2.2030(15)	O2-Yb1-O1	90.44(5)	
Yb1-O1	2.2952(14)	O2-Yb1-O1W	89.70(6)	
Yb1-O1W	2.3074(16)	O1-Yb1-N3	143.47(5)	
Yb1-O2W	2.3378(14)	O1W-Yb1-O2W	145.66(6)	
Yb1-O3W	2.3578(15)	O1W-Yb1-N2	81.05(6)	
Yb1-N1	2.4230(18)	O1-Yb1-N2	127.43(6)	
Yb1-N2	2.4263(18)	N1-Yb1-N2	65.75(7)	
Yb1-N3	2.4581(19)	N1-Yb1-N3	130.75(6)	
D-H...A	d(D-H)	d(H...A)	d(D...A)	<(DHA)
O1W-H1W1...O4W	0.78	2.07	2.747(2)	145.2
O2W-H2W2...O2[#1]	0.86	1.78	2.638(2)	171.5
O2W-H2W1...O1S[#2]	0.91	2.16	3.040(2)	163.9
O3W-H3W1...O1[#1]	0.89	1.77	2.654(2)	172.4
O3W-H3W2...O1S	0.89	1.89	2.755(2)	165.3
O4W-H4W1...O3W[#5]	0.88	2.60	3.333(2)	141.5
O4W-H4W1...O2[#2]	0.88	2.29	2.997(2)	136.9
O4W-H4W2...O1	0.84	2.14	2.863(2)	143.4
O1W-H1W1...O4W	0.78	2.07	2.747(2)	145.2

[#1] -x+1/2,y-1/2,-z+1/2; [#2] -x+1/2,y+1/2,-z+1/2; [#5] x,y+1,z

Table S4. SHAPE v2.1. Continuous Shape Measures Calculation (c) 2013 Electronic Structure Group, Universitat de Barcelona.

Coordination number 7

HP-7	1 D7h	Heptagon
HPY-7	2 C6v	Hexagonal pyramid
PBPY-7	3 D5h	Pentagonal bipyramid
COC-7	4 C3v	Capped octahedron
CTPR-7	5 C2v	Capped trigonal prism
JPBY-7	6 C5h	Johnson pentagonal bipyramid J13
JETPY-7	7 C3v	Johnson elongated triangular pyramid J7

Coordination number 8

OP-8	1 D8h	Octagon
HPY-8	2 C7v	Heptagonal pyramid
HBPY-8	3 D6h	Hexagonal bipyramid
CU-8	4 Oh	Cube
SAPR-8	5 D4d	Square antiprism
TDD-8	6 D2d	Triangular dodecahedron
JGBF-8	7 D2d	Johnson gyrobifastigium J26
JETBPY-8	8 D3h	Johnson elongated triangular bipyramid J14
JBTPR-8	9 C2v	Biaugmented trigonal prism J50
BTPR-8	10 C2v	Biaugmented trigonal prism
JSD-8	11 D2d	Snub diphenoid J84
TT-8	12 Td	Triakis tetrahedron
ETBPY-8	13 D3h	Elongated trigonal bipyramid

1·CH_3OH

Structure [ML_7]	HP-7	HPY-7	**PBPY-7**	COC-7	CTPR-7	JPBPY-7	JETPY-7
	34.355,	26.096,	**0.986,**	8.929,	7.340,	5.848,	24.501

2·CH_3CH_2OH·H_2O

Structure [ML_8]	ETBPY-8	TT-8	JSD-8	BTPR-8	JBTPR-8	JETBPY-8
	22.267,	13.810,	2.970,	2.166,	2.729,	25.511,

JGBF-8	**TDD-8**	SAPR-8	CU-8	OP-8	HPY-8	HBPY-8
11.147,	**1.410**,	3.835,	13.255,	15.365,	31.785	23.137

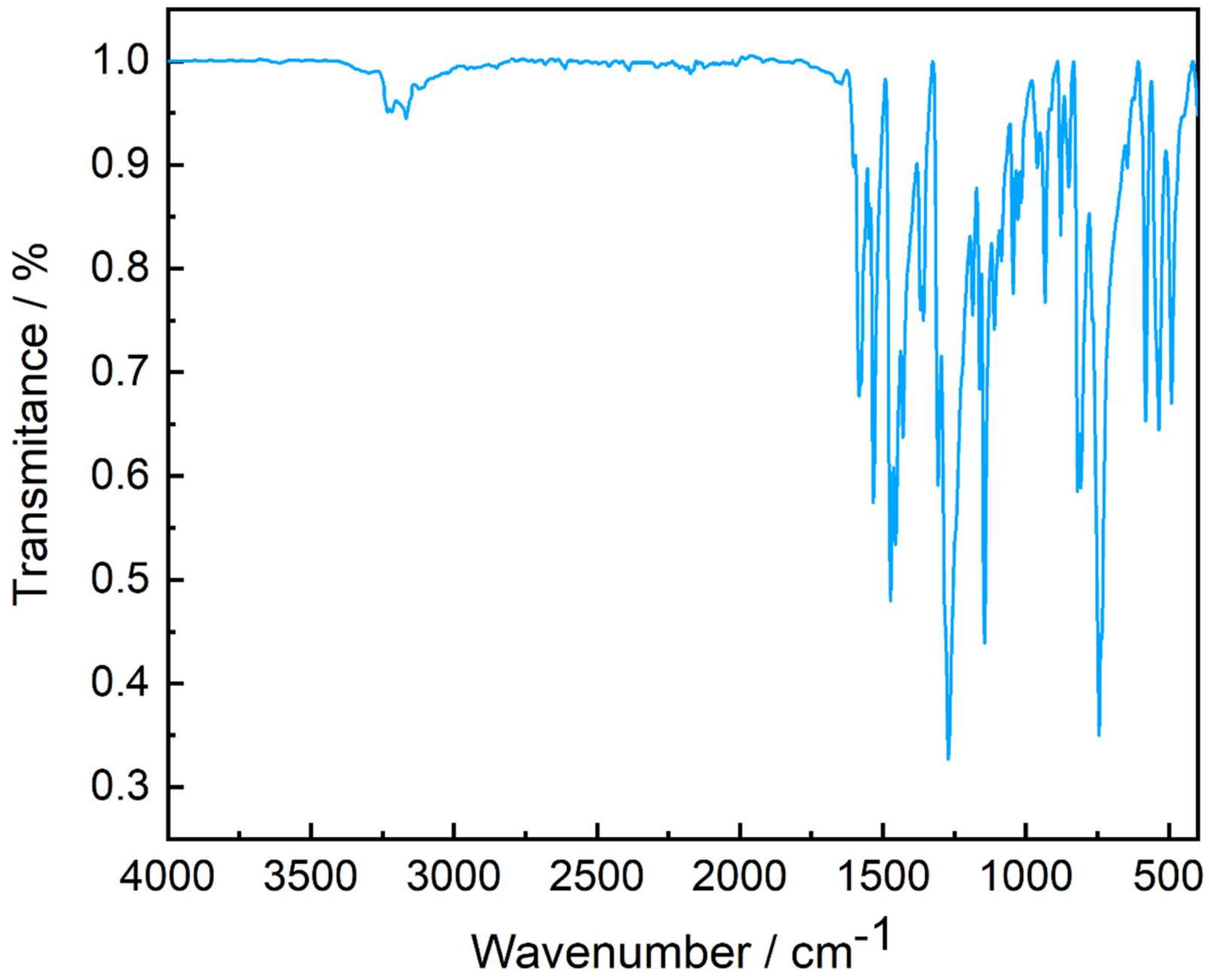

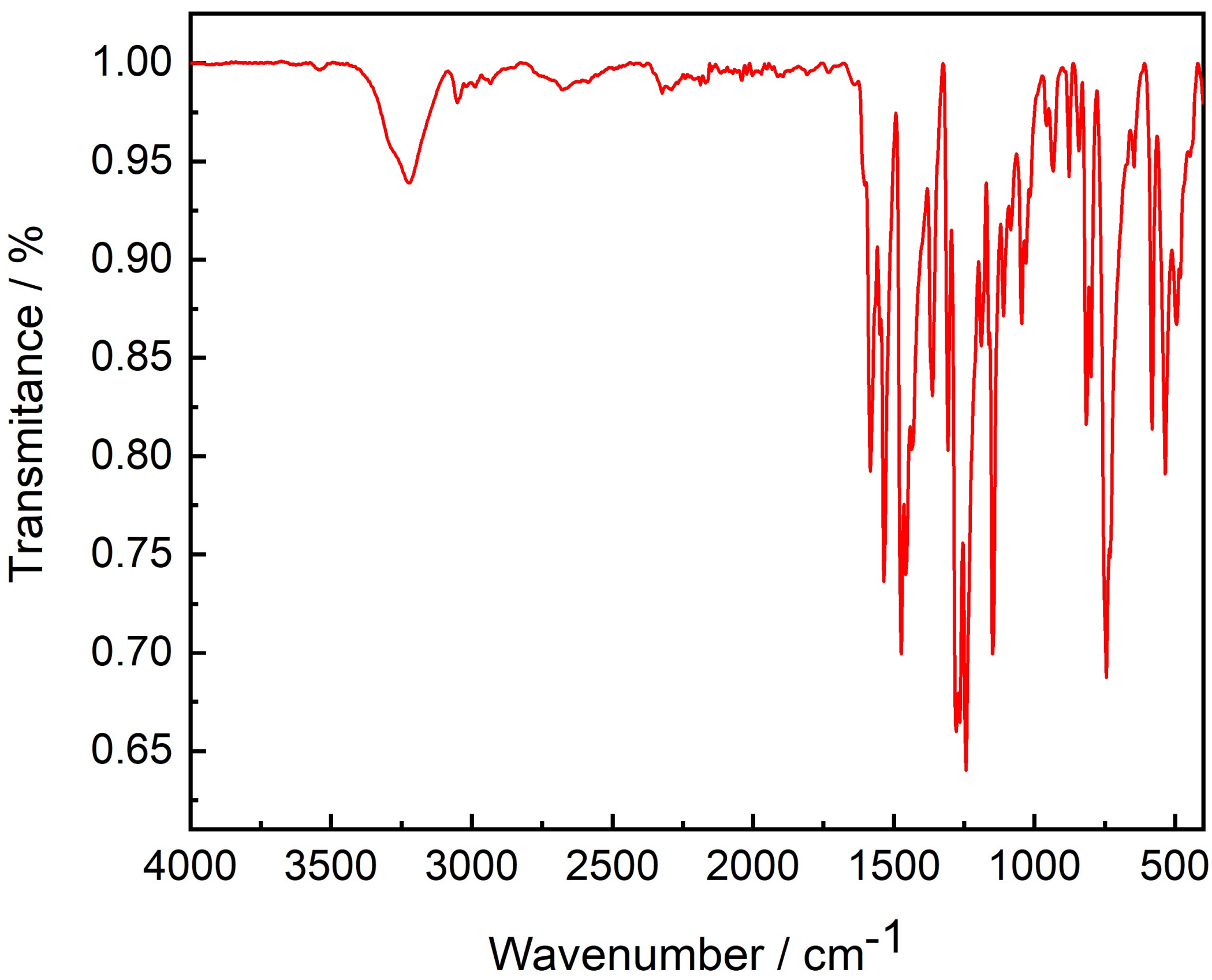

Figure S1. IR spectra of **1** (up) and **2**·CH_3CH_2OH·H_2O (bottom).

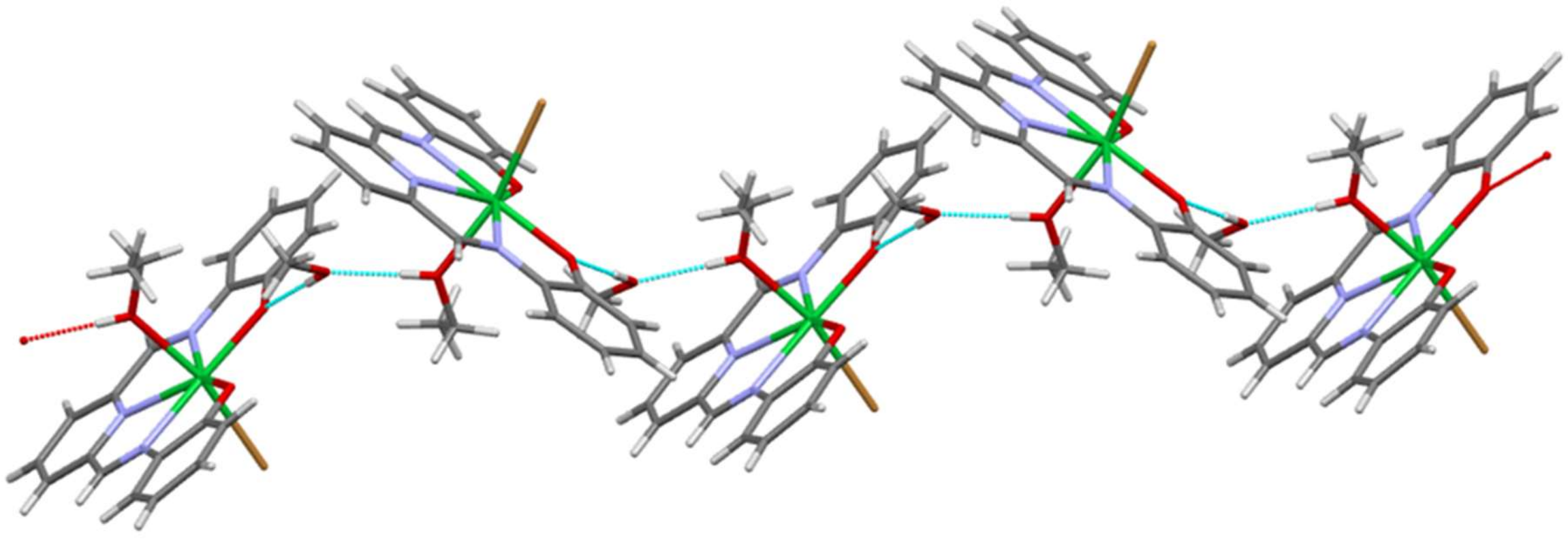

Figure S2. Balls and sticks diagram for **1**·CH_3OH illustrating the zigzag chain structure stabilized by intermolecular hydrogen bonds.

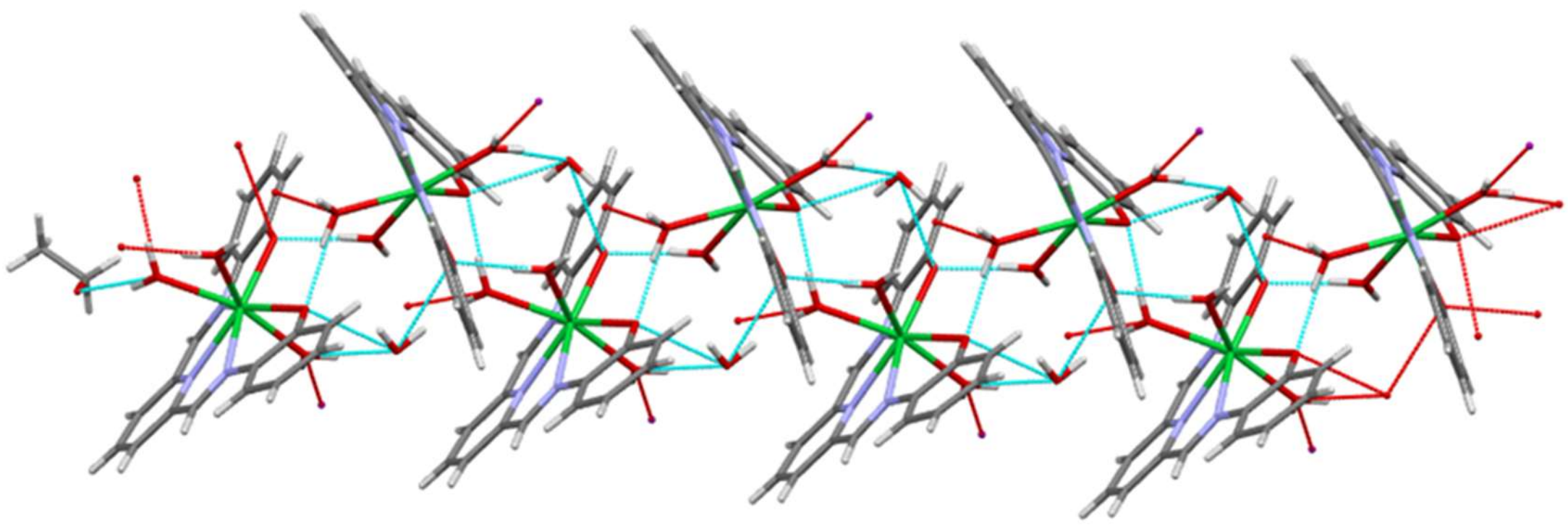

Figure S3. Balls and sticks diagram for **2**·CH_3CH_2OH·H_2O illustrating the zigzag chain structure stabilized by intermolecular hydrogen bonds.

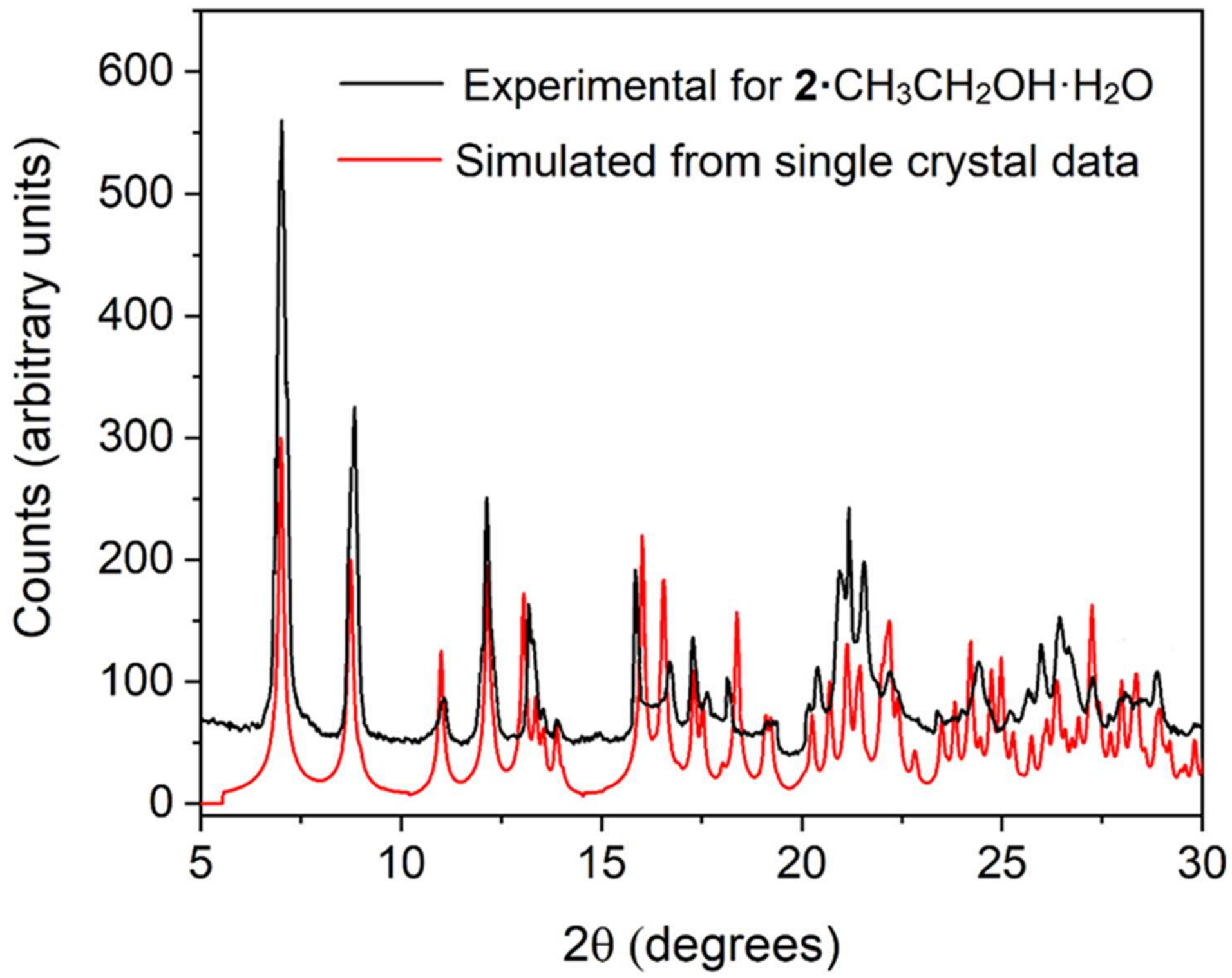

Figure S4. Comparative powder X-ray diffractograms for **2**·CH_3CH_2OH·H_2O (black) and the simulation from single X-ray diffraction data (red).

2. MAGNETIC CHARACTERIZATION

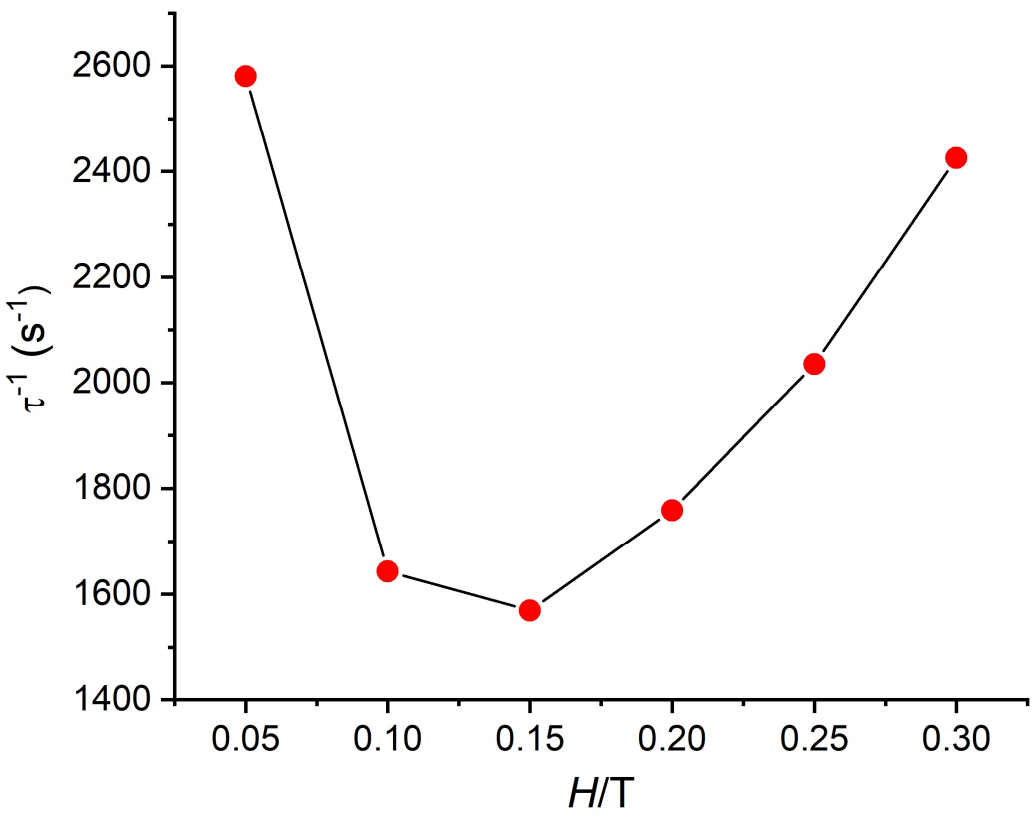

Figure S5. Dependence of the relaxation time with the field for **1** at 3 K. The solid line is a guide for the eyes.

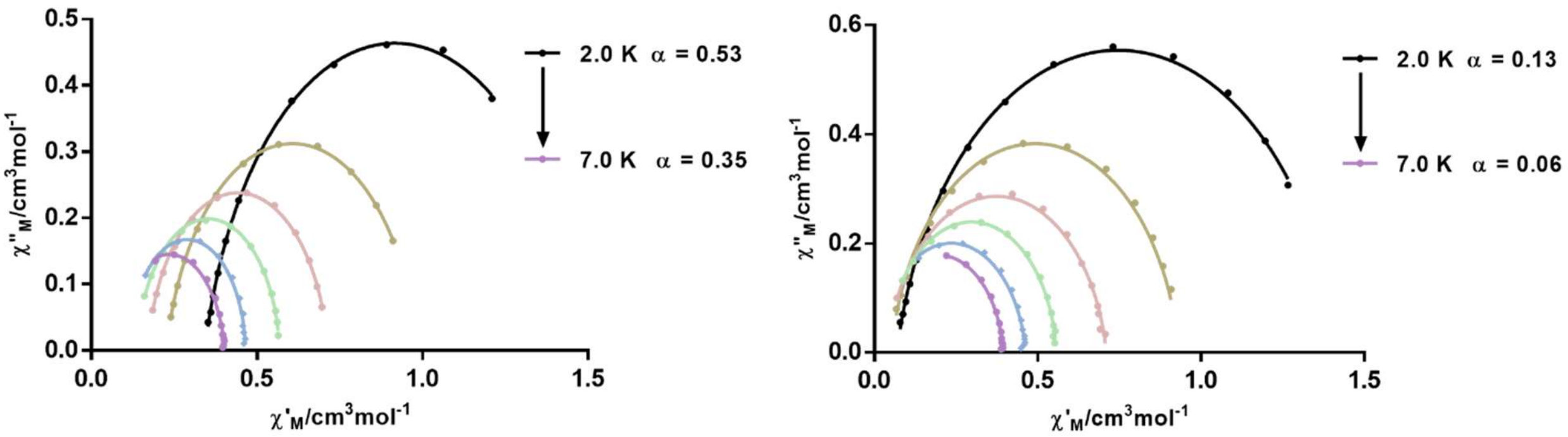

Figure S6. Cole Cole plots for **1** (left) and **2**·CH_3CH_2OH·H_2O (right) in H_{dc} = 1500 Oe.

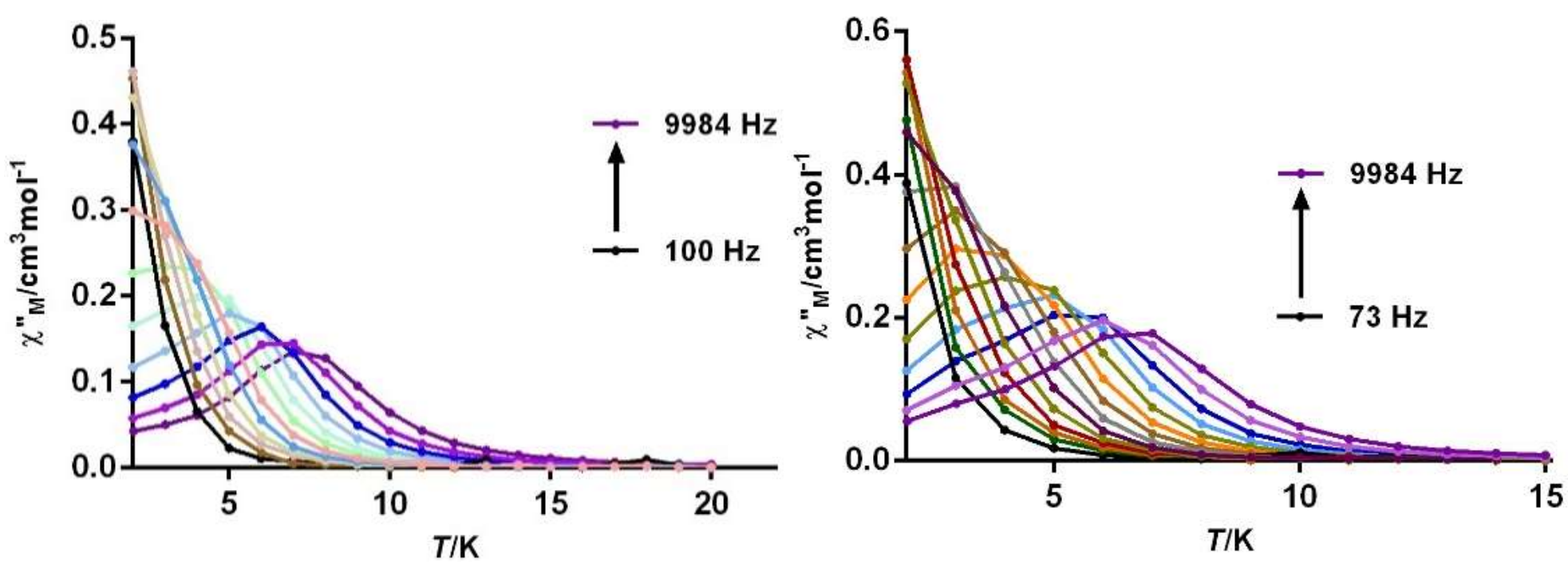

Figure S7. Temperature dependence of χ''_M for **1**·CH_3OH (left) and **2**·CH_3CH_2OH·H_2O (right) in H_{dc} = 1500 Oe at different frequencies. The solid lines are guides for the eyes.

3. SPECTROSCOPIC CHARACTERIZATION

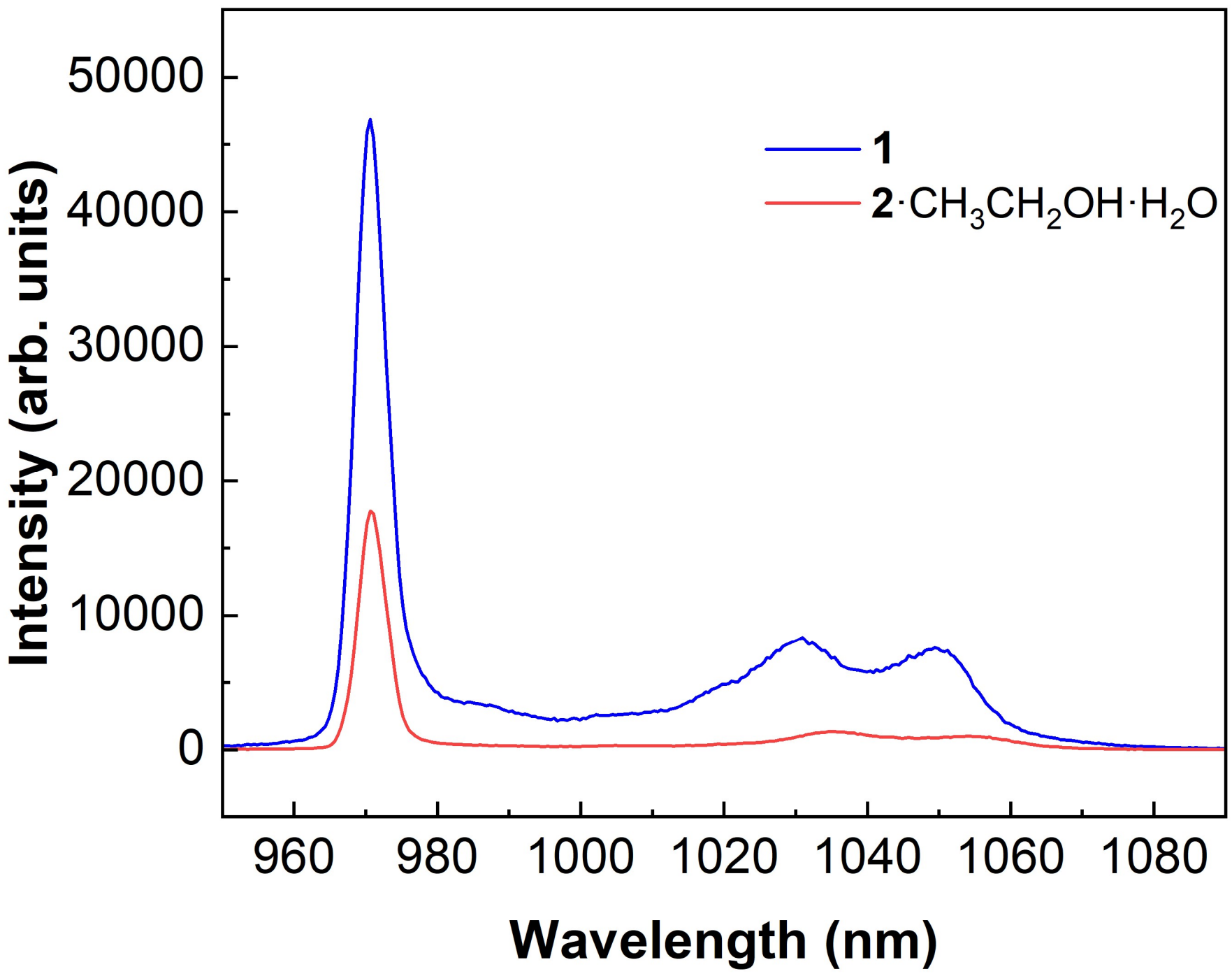

Figure S8. Room-temperature emission spectra of complexes **1** and **2**·$CH_3CH_2OH{\cdot}H_2O$ recorded under excitation at 325 nm.

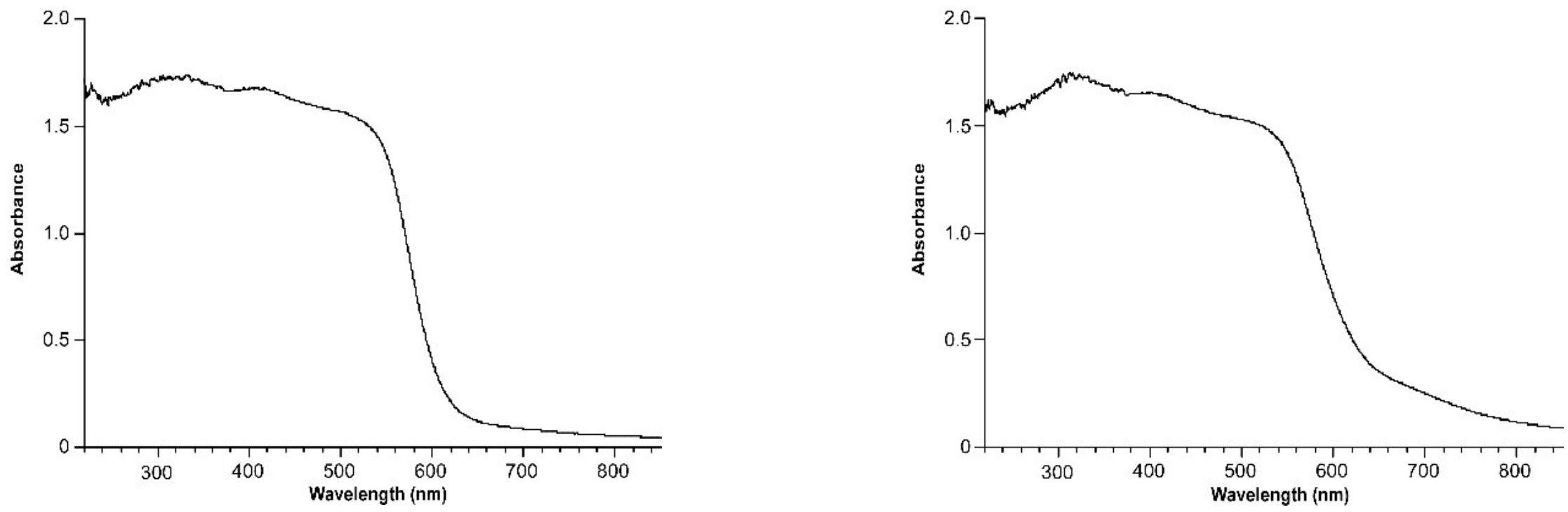

Figure S9. Solid state UV-Vis absorption spectra for compounds **1** and **2**·$CH_3CH_2OH{\cdot}H_2O$.

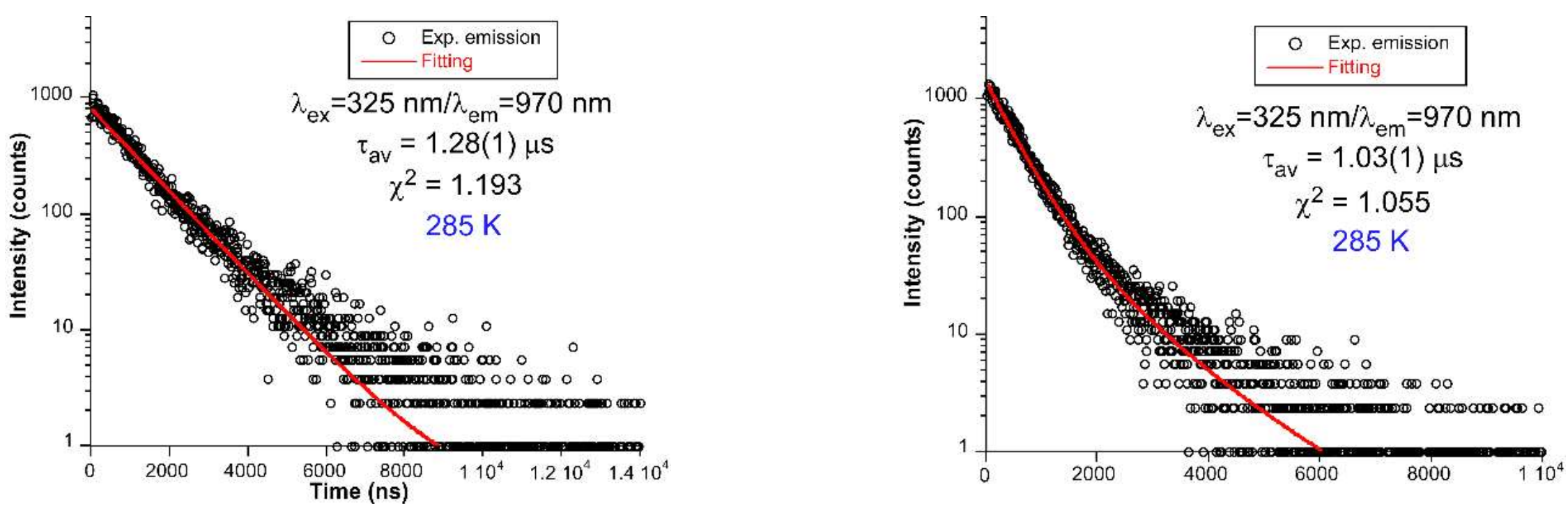

Figure S10. Comparative analysis of the decay curves of the Yb-centered NIR emission under UV illumination (λ_{ex} = 325 nm) for compounds **1** (left) and **2**·$CH_3CH_2OH{\cdot}H_2O$ (right). Note that tail fitting was employed to estimate the lifetimes.

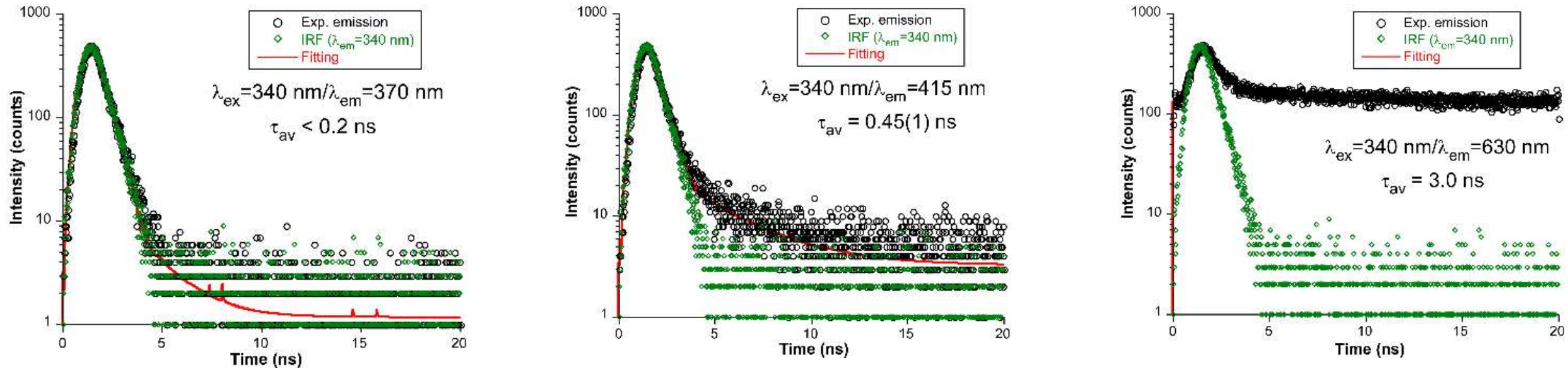

Figure S11. Decay curves of the ligand-centered emission components taken at room temperature under UV-LED illumination (λ_{ex} = 340 nm) on a powdered polycrystalline sample of **1**. Note that reconvolution fitting was employed to estimate the lifetimes owing to the short life of the processes, which are close to the pulse of the LED.

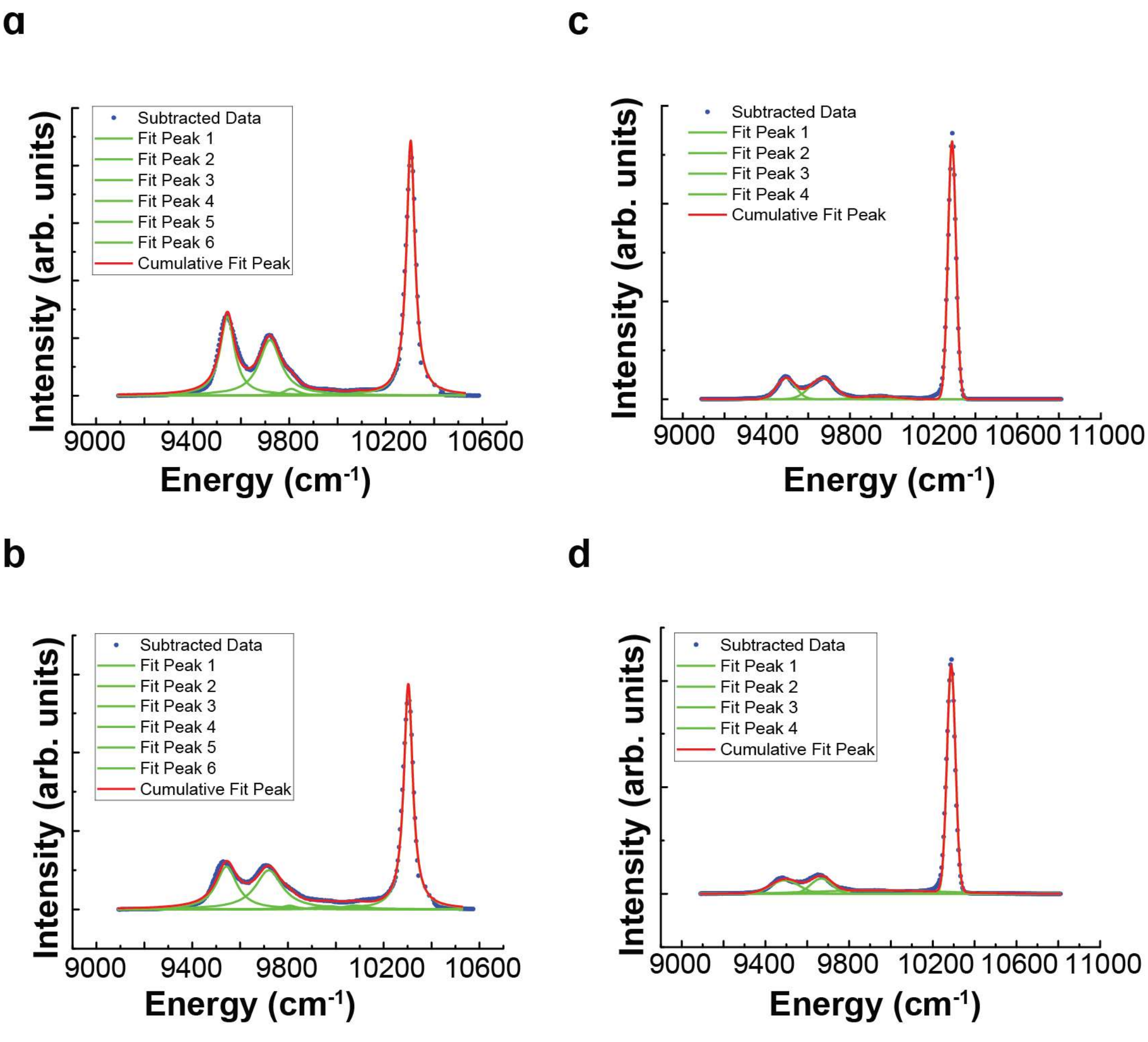

Figure S12. Temperature-dependent emission spectra and spectral deconvolution of compounds **1** and **2**·$CH_3CH_2OH{\cdot}H_2O$ recorded at 15 and 210 K upon excitation at 325 nm**.** Panels **(a)** and **(b)** correspond to compound **1** at 15 and 210 K, respectively, whereas panels **(c)** and **(d)** show compound **2**·$CH_3CH_2OH{\cdot}H_2O$ at 15 and 210 K**,** respectively. The blue circles represent the experimental data, while the **red line** corresponds to the overall fit obtained using multiple Gaussian functions.

Table S5. Energy of the Kramers doublets (KDs) obtained from spectroscopic measurements of complexes **1** and **2**·$CH_3CH_2OH{\cdot}H_2O$

Energy (cm^{-1})			
		**1**	**2**·$CH_3CH_2OH{\cdot}H_2O$
$^2F_{7/2}$	KD1	0	0
	KD2	496.54	353.92
	KD3	582.82	620.99
	KD4	759.50	793.87
$^2F_{5/2}$	KD1	10302.86	10288.074
	KD2	10728.23	-

4. Ab Initio CALCULATIONS

Table S6. Energies of the Kramers doublets within the ground state of **1** and **2**.

Energy (cm^{-1})							
		**1**			**2**		
$^2F_{7/2}$		CASSCF	NEVPT2	QD-NEVPT2	CASSCF	NEVPT2	QD-NEVPT2
	KD1	0	0	0	0	0	0
	KD2	487.26	622.88	608.02	481.12	596.15	595.10
	KD3	596.42	730.91	730.23	631.93	787.91	776.73
	KD4	666.51	845.33	831.05	775.22	966.80	962.32

Table S7. Components of the g-tensor and tilting angle (θ) of the eigenvector associated to the g_z component of the corresponding excited state KD with respect to the ground state KD for **1**, derived from CASSCF calculations.

	g_x	g_y	g_z	θ (°)
KD1	0.9372	1.0722	7.4950	
KD2	2.1265	2.8518	4.6479	96.1
KD3	0.2474	1.1630	6.4399	90.3
KD4	0.8497	1.1337	6.6517	88.5

Table S8. Components of the g-tensor and tilting angle (θ) of the eigenvector associated to the g_z component of the corresponding excited state KD with respect to the ground state KD for **2** derived from CASSCF calculations.

	g_x	g_y	g_z	θ (°)
KD1	0.8597	1.1987	7.4906	
KD2	1.0552	2.4099	4.6849	14.6
KD3	1.6229	1.8581	6.0288	82.7
KD4	0.4455	0.7949	7.5367	86.0

5. LUMINESCENCE THERMOMETRY

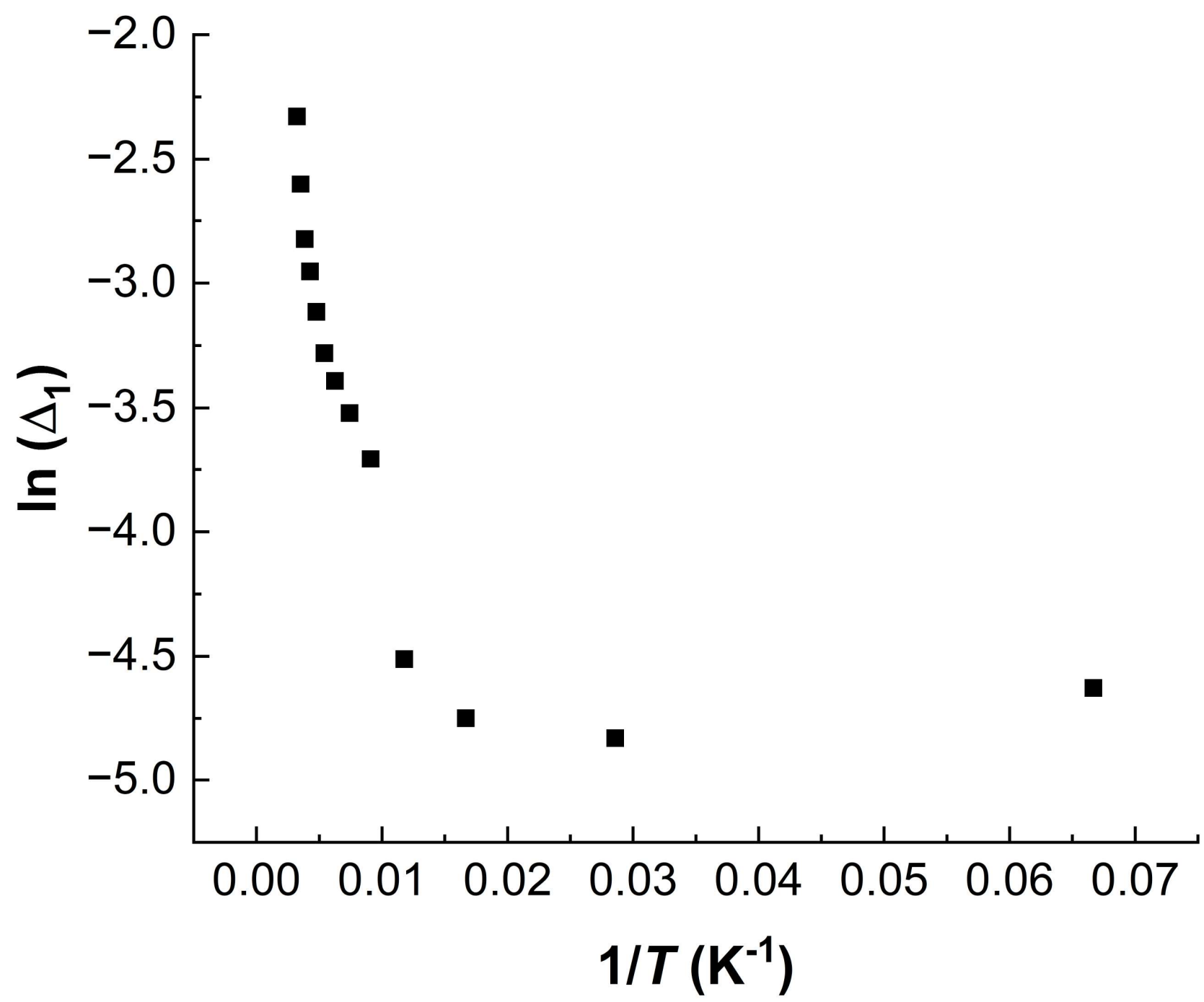

Figure S13. Temperature dependence of ln(Δ_1) as a function of inverse temperature (1/*T*) in **1**.

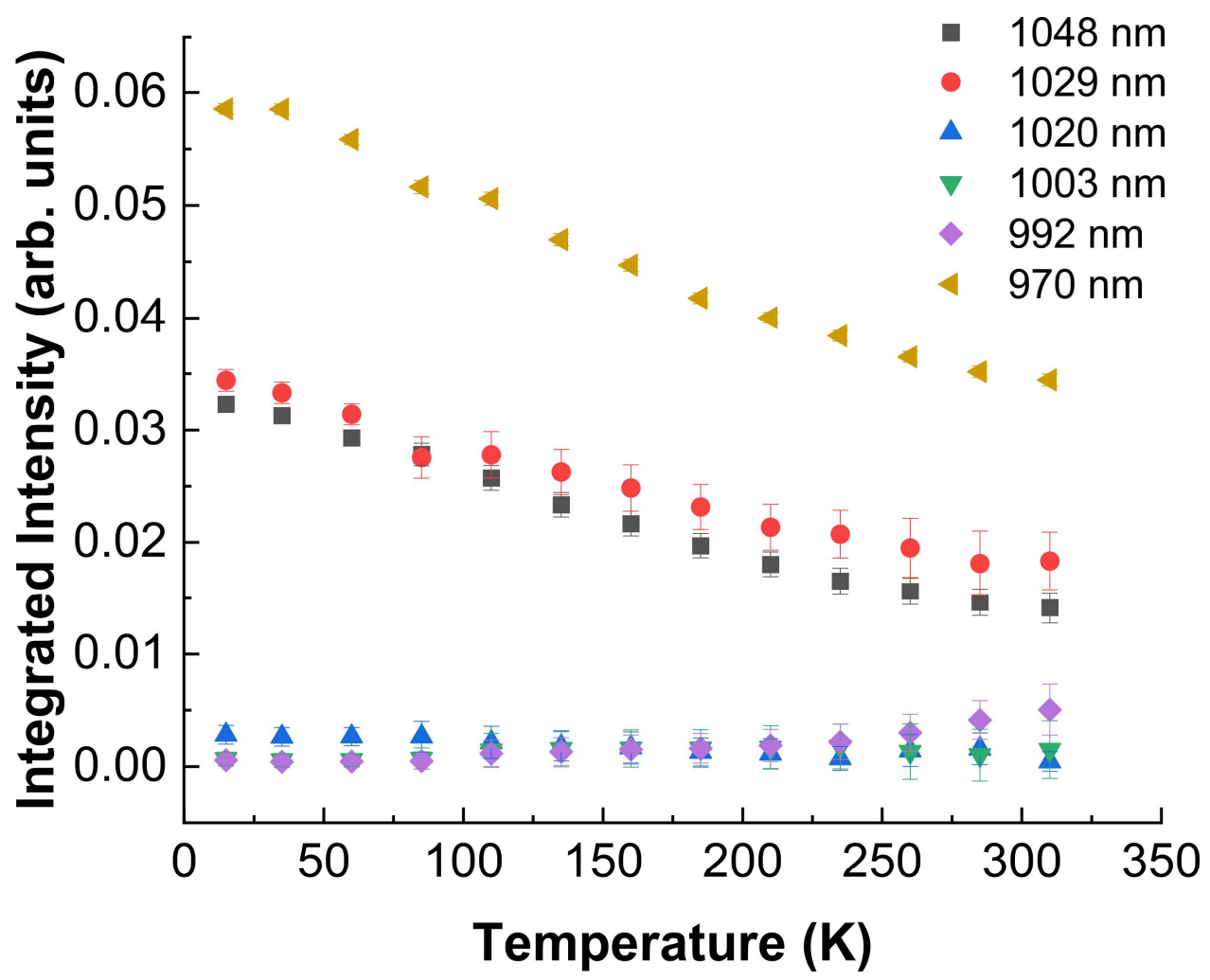

Figure S14. Temperature-dependent of the integrated Stark component areas for the Yb^{3+} $^2F_{5/2}\rightarrow{}^2F_{7/2}$ transition in **1**.

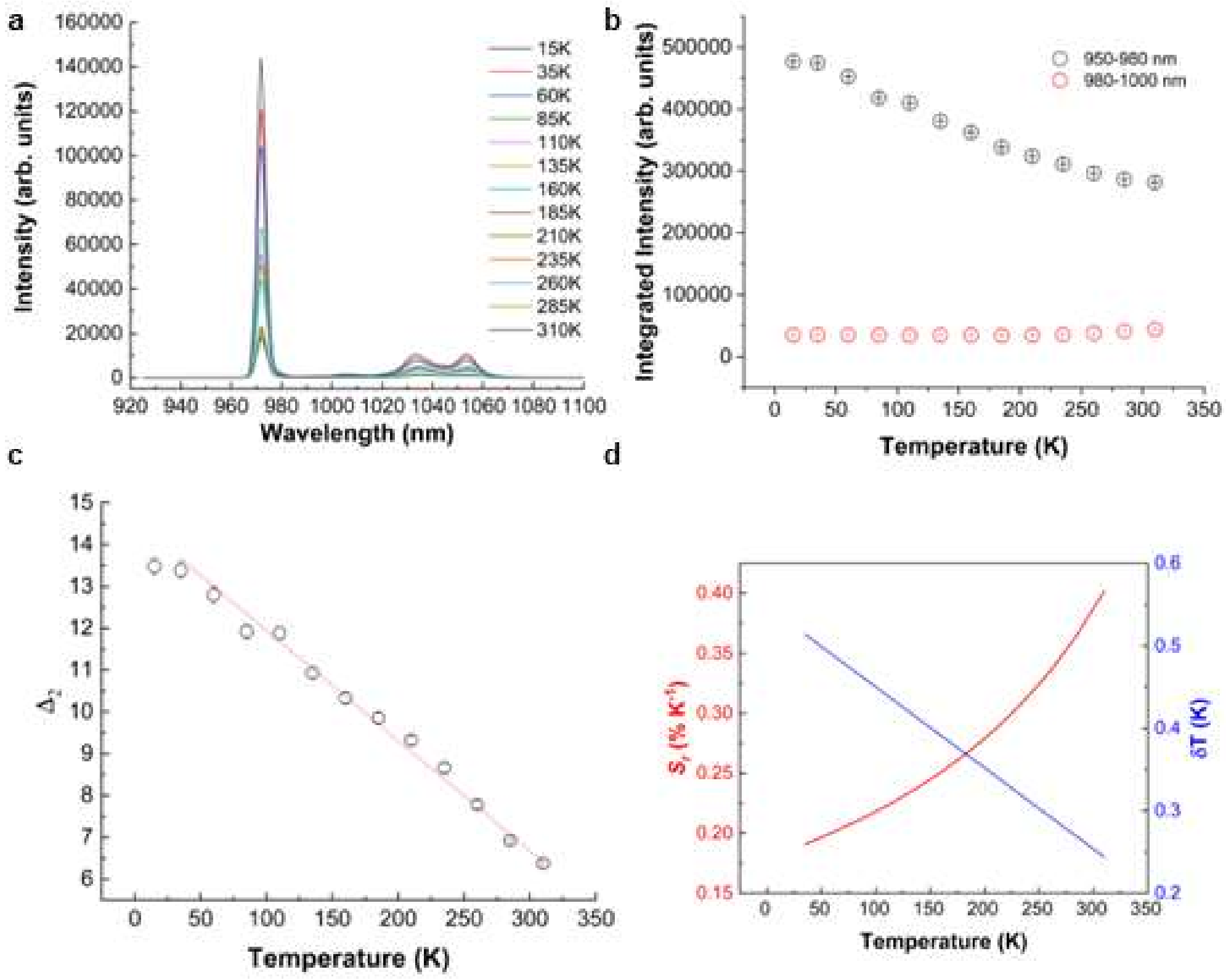

Figure S15. (a) Temperature-dependent Yb^{3+} $^2F_{5/2}\rightarrow{}^2F_{7/2}$ transition in the NIR emission spectra of complex **2**·$CH_3CH_2OH{\cdot}H_2O$ in the 15-310 K temperature range. (b) Temperature evolution of the integrated intensities in the 950-980 and 980-1000 nm spectral regions (c) Temperature dependence of the thermometric parameter $\boldsymbol{\Delta_2}$. The solid line represents the best fit of the experimental data to a linear function (fit details in Table S9). (d) Temperature dependence of the relative sensitivity ($\boldsymbol{S_r}$) and temperature uncertainty ($\boldsymbol{\delta T}$) associated with $\boldsymbol{\Delta_2}$

Table S9. Summary of the fitting parameters for the plots of complexes **1** and **2**·$CH_3CH_2OH{\cdot}H_2O$

Plot	Linear	a	b	r^2	Operating range (K)
Figure 10c	y = a + bx	-0.0090 ± 0.0029	(2.70 ± 0.15)×10^{-4}	0.983	60-310
Figure S15c		14.58 ± 0.18	(-26.38 ± 0.69)×10^{-3}	0.993	35-310

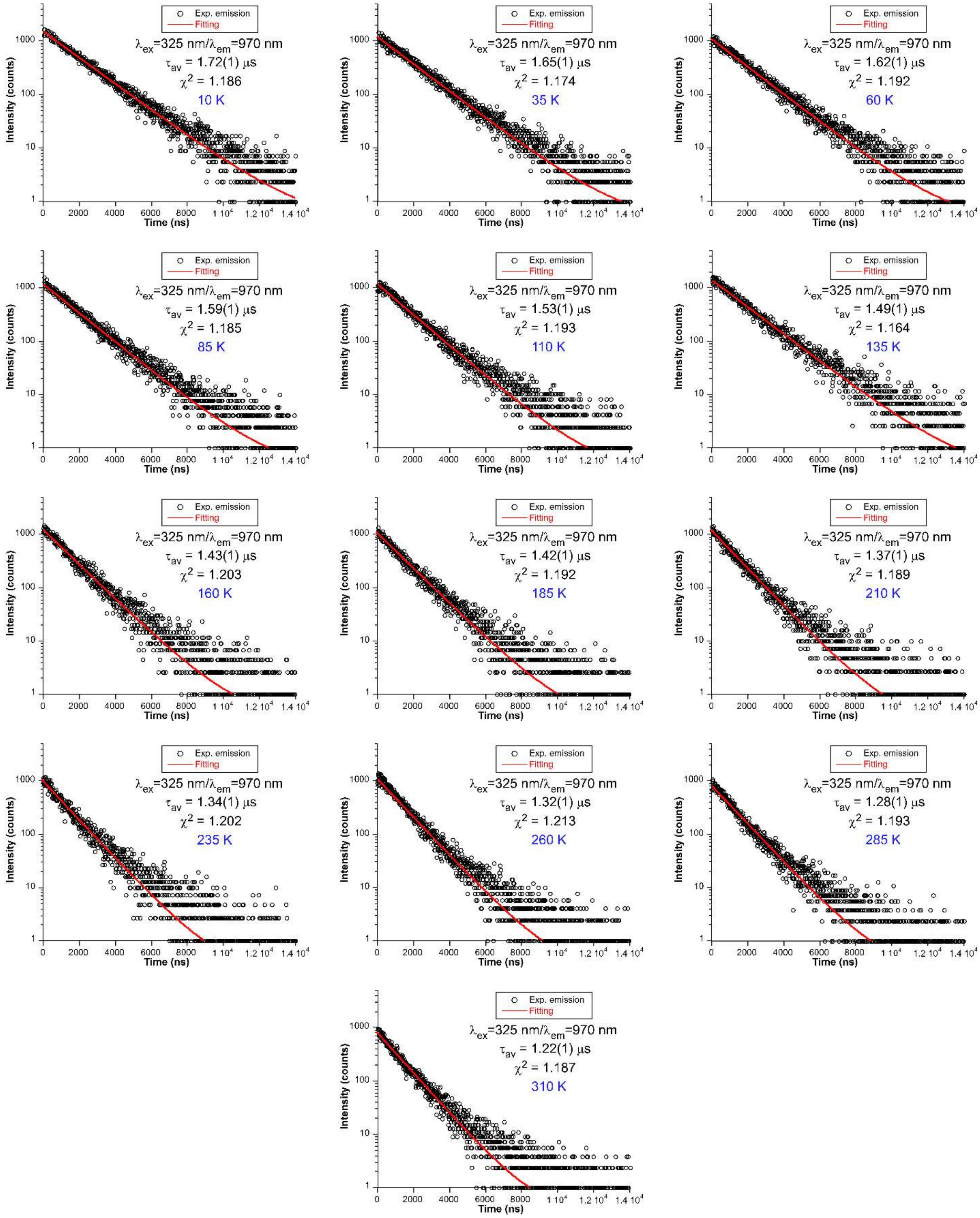

Figure S16. Variable-temperature decay curves of the Yb-centered NIR emission under UV illumination (λ_{ex} = 325 nm) on a powdered polycrystalline sample of **1**. Note that tail fitting was employed to estimate the lifetimes.

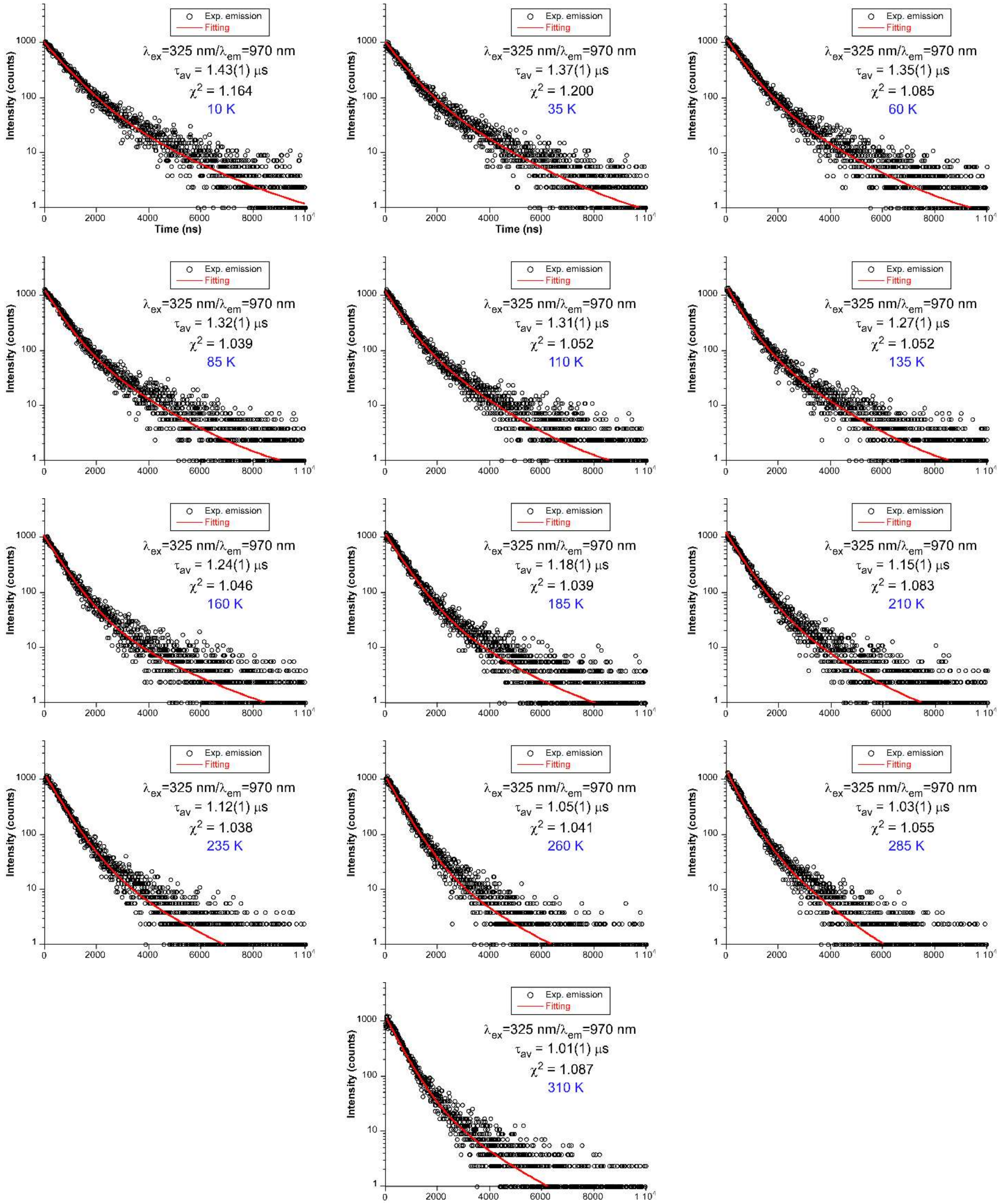

Figure S17. Variable-temperature decay curves of the Yb-centered NIR emission under UV illumination (λ_{ex} = 325 nm) on a powdered polycrystalline sample of **2**$\cdot CH_3CH_2OH\cdot H_2O$. Note that tail fitting was employed to estimate the lifetimes.

Table S10. Excitation wavelength (λ), temperature range (*ΔT*), maximum relative sensitivity (S_m), and corresponding temperature (T_m) of the Yb^{3+}-based thermometers with slow relaxation of the magnetization reported so far.

Yb^{+3}-SMM thermometer	U_{eff} (K) / H_{dc} (KOe)	λ (nm)	ΔT (K)	S_m (% K^{-1})	T_m (K)	Ref.
$[Yb(L)Br(CH_3CH_2OH)]$	Not Orbach /1.5	325	60-310	3.7	60	This work
$[Yb(L)(H_2O)_3]I\cdot CH_3CH_2OH\cdot H_2O$	Not Orbach /1.5	325	35-310	0.4	310	This work
$[Yb(H_3L^{1,1,4})]\cdot 2MeOH$	Not Orbach / 1.6	360	25-300	0.2	300	1
$[Yb(TPPO)_3(NCS)_3]$	nr/1.5	320	10–300	1.5	150	2
$[Yb(TPPO)_3(NCSe)_3]$	nr/1.5	360	10–300	1.7	150	
$\{(H_5O_2)_2(H)[Yb(hmpa)_4][Co(CN)_6]_2\}$	Not Orbach / 1.0	320	50-175	4.6	50	3
$[YbCo(CN)_6(bpyO_2)_2(H_2O)_3]$	Not Orbach / 1.25	315	125-275	0.1	180	4
$[Nd_{0.757}Yb_{0.243}(2,2'\text{-bpdo})_2(H_2O)][Cu_2(CN)_5]\cdot 5H_2O$	Not Orbach / 2	370	50-155	2.6	50	5
$[Nd_{0.509}Yb_{0.491}(2,2'\text{-bpdo})_2(H_2O)][Cu_2(CN)_5]\cdot 5H_2O$	Not Orbach / 1	370	50-220	3.4	50	5
$[Nd_{0.228}Yb_{0.772}(2,2'\text{-bpdo})_2(H_2O)][Cu_2(CN)_5]\cdot 5H_2O$	Not Orbach / 1	370	50-275	2.3	130	5
$[Yb(2,2'\text{-bpdo})_2(H_2O)][Cu_2(CN)_5]\cdot 5H_2O$	Not Orbach / 1	370	75-250	5.1	75	5
$[Yb_2(valdien)_2(NO_3)_2]$	65.6 /1.0	390	80–320	1.1	130	6

$H_3L^{1,1,4}$ = N_4O_3 aminophenol; $bpyO_2$ = 2,2′-dipyridyl N,N′-dioxide; H_2valdien = N1,N3-bis(3-methoxysalicylidene)diethylenetriamine; TPPO = triphenylphosphine oxide; hmpa = hexamethylphosphoramide; 2,2′-bpdo = 2,2′-bipyridine *N*,*N*′-dioxide

REFERENCES

(1) Fondo, M.; Corredoira-Vázquez, J.; García-Deibe, A. M.; Sanmartín-Matalobos, J.; Amoza, M.; Botas, A. M. P.; Ferreira, R. A. S.; Carlos, L. D.; Colacio, E. Field-induced slow magnetic relaxation and luminescence thermometry in a mononuclear ytterbium complex. *Inorg. Chem. Front.* **2020**, *7* (16), 3019-3029. DOI: http://dx.doi.org/10.1039/D0QI00637H.

(2) Kumar, K.; Stefanczyk, O.; Nakabayashi, K.; Imoto, K.; Oki, Y.; Ohkoshi, S.-i. Detection of sub-terahertz Raman response and nonlinear optical effects for luminescent Yb(III) complexes. *Adv. Optical Mater.* **2022**, *10* (2), 2101721. DOI: https://doi.org/10.1002/adom.202101721.

(3) Wang, J.; Zakrzewski, J. J.; Heczko, M.; Zychowicz, M.; Nakagawa, K.; Nakabayashi, K.; Sieklucka, B.; Chorazy, S.; Ohkoshi, S.-i. Proton conductive luminescent thermometer based on near-infrared emissive $\{YbCo_2\}$ molecular nanomagnets. *J. Am. Chem. Soc.* **2020**, *142* (8), 3970-3979. DOI: https://doi.org/10.1021/jacs.9b13147.

(4) Karachousos-Spiliotakopoulos, K.; Tangoulis, V.; Panagiotou, N.; Tasiopoulos, A.; Moreno-Pineda, E.; Wernsdorfer, W.; Schulze, M.; Botas, A. M. P.; Carlos, L. D. Luminescence thermometry and field induced slow magnetic relaxation based on a near infrared emissive heterometallic complex. *Dalton Trans.* **2022**, *51* (21), 8208-8216. DOI: http://dx.doi.org/10.1039/D2DT00936F.

(5) Xin, Y.; Kumar, K.; Zakrzewski, J. J.; Chorazy, S.; Nakabayashi, K.; Stefanczyk, O.; Ohkoshi, S.-i. Tuning of ratiometric optical thermometry in the family of magnetic $\{Nd_{1-x}{}^{III}Yb^{III}{}_x[Cu_2I(CN)_5]\}$ frameworks showing the large SHG response. *J. Phys. Chem. C* **2023**, *127* (31), 15500-15511. DOI: https://doi.org/10.1021/acs.jpcc.3c02105.

(6) Brunet, G.; Marin, R.; Monk, M.-J.; Resch-Genger, U.; Gálico, D. A.; Sigoli, F. A.; Suturina, E. A.; Hemmer, E.; Murugesu, M. Exploring the dual functionality of an ytterbium complex for luminescence thermometry and slow magnetic relaxation. *Chem. Sci.* **2019**, *10* (28), 6799-6808. DOI: http://dx.doi.org/10.1039/C9SC00343F.
